# A detailed investigation of the visual system and visual ecology of the Barrier Reef anemonefish, *Amphiprion akindynos*

**DOI:** 10.1038/s41598-019-52297-0

**Published:** 2019-11-11

**Authors:** Sara M. Stieb, Fanny de Busserolles, Karen L. Carleton, Fabio Cortesi, Wen-Sung Chung, Brian E. Dalton, Luke A. Hammond, N. Justin Marshall

**Affiliations:** 1 Queensland Brain Institute, The University of Queensland, Brisbane, QLD 4072 Australia; 2 Centre of Ecology, Evolution and Biogeochemistry, EAWAG Swiss Federal Institute of Aquatic Science and Technology, Kastanienbaum, Switzerland; 3 Institute of Ecology and Evolution, University of Bern, Bern, Switzerland; 4 Department of Biology, University of Maryland, College Park, MD 20742 USA

**Keywords:** Sensory processing, Transcriptomics, Evolutionary ecology

## Abstract

Vision plays a major role in the life of most teleosts, and is assumingly well adapted to each species ecology and behaviour. Using a multidisciplinary approach, we scrutinised several aspects of the visual system and ecology of the Great Barrier Reef anemonefish, *Amphiprion akindynos*, including its orange with white patterning, retinal anatomy and molecular biology, its symbiosis with anemones and sequential hermaphroditism. *Amphiprion akindynos* possesses spectrally distinct visual pigments and opsins: one rod opsin, RH1 (498 nm), and five cone opsins, SWS1 (370 nm), SWS2B (408 nm), RH2B (498 nm), RH2A (520 nm), and LWS (554 nm). Cones were arranged in a regular mosaic with each single cone surrounded by four double cones. Double cones mainly expressed *RH2B* (53%) in one member and *RH2A* (46%) in the other, matching the prevailing light. Single cones expressed *SWS1* (89%), which may serve to detect zooplankton, conspecifics and the host anemone. Moreover, a segregated small fraction of single cones coexpressed *SWS1* with *SWS2B* (11%). This novel visual specialisation falls within the region of highest acuity and is suggested to increase the chromatic contrast of *Amphiprion akindynos* colour patterns, which might improve detection of conspecifics.

## Introduction

Vision plays a major role in the life of most teleost fishes to enable foraging, avoidance of predators, navigation, and mate choice (e.g., reviewed in^1–3^). Visual tasks may differ between species and individuals, and also depend on the light habitat or environment.

Visual adaptations may be found at the optical level. For example, filters present in the lens or cornea may block specific wavelengths of light, and thus indirectly shape an organism’s visual sensitivity before light reaches the retina^4–6^.

At the retinal level, the main adaptations are found in the contents of photoreceptors and the interconnections of ganglion cells. Photoreceptors are composed of rod and cone cells^7^ that house light-sensitive visual pigments^8^. Rods possess the highly sensitive rod opsin-based photopigment (RH1) and mediate vision in scotopic conditions. Cones contain up to four cone opsin-based photopigments being short- (SWS1 and SWS2), medium- (RH2) and long-wavelength-sensitive (LWS)^8^, are active in photopic conditions, and mediate colour vision. Teleost cones can further be divided based on their morphology into single cones and double or twin cones (i.e., two single cones fused together)^9^. Ganglion cells directly send the visual information to the central nervous system and their receptive field ultimately set the upper limit of spatial resolving power or visual acuity^10^.

The type, composition, arrangement and density of both photoreceptor and ganglion cells varies between species (interspecific variability) as well as across the retina of a single species (intraretinal variability), and has been shown to be directly linked to a species’ ecology and behaviour^3,11–14^. Interspecific variability in the number and type of photoreceptor cells correlates well with the light habitat and lifestyle of a species. Nocturnal species, for example, have more rods than diurnal ones and most deep-sea species have completely lost their cones in favour of a rod-only retina^13^. Conversely, diurnal coral reef fishes that live in a very bright and colourful environment, often have acute colour vision provided by complex photoreceptor mosaics of several cone types being sensitive to a wide spectrum of light^15^. Also, interspecific variability in the number and connectivity of ganglion cells, and therefore the visual acuity, often reflects the feeding behaviour of a species^10^.

Intraretinal variability is often assessed by constructing topographic maps of photoreceptor and ganglion cell distribution. This allows the identification of regions of high-cell densities or specialisations, which provide a higher sensitivity or acuity in a specific part of the visual field of the animal. The retinal topography usually reflects the structure of the habitat (terrain theory^14,16^), the behavioural ecology (e.g., feeding strategy^17^, predator avoidance^18^), the light environment^10^, and the life stage of an animal^19^.

At the molecular level, opsin genes have evolved by gene duplication^20^, deletion and conversion (e.g.,^21^), by mutation of the opsin sequence itself (e.g.,^22^), and by variability in opsin gene expression^23^. Changes in opsin gene repertoire and expression have been found to facilitate visual system adaptation to the prevailing light habitat (e.g.,^24–29^), predation density^30^ and/or feeding strategies^25,31^. This might also include intraretinal differences in opsin gene expression to optimise vision in different directions^11^. Recent research has shown that the coexpression of spectrally distinct opsin genes in double^32^ and single^33^ cones, together with intraretinal differences in distribution, may enable concise spectral tuning of the visual field to increase the detection of prey, predators and mates.

Damselfishes (Pomacentridae) are particularly well-suited to understand the mechanisms underlying visual system adaptation. With more than 390 species, they are an abundant, colourful, and diverse coral reef fish family^34^. They also possess some of the widest ranges of spectral sensitivities in the marine realm; species having three to four distinct visual pigments ranging in sensitivity from ultraviolet (UV) to long-wavelengths^15,31^. The anemonefishes (sub-family, Amphiprioninae) form a unique group within the damselfishes. They are famous not only for their highly contrasting colour patterns^15,35,36^, but also for their specialised ecology and life cycle. Besides living in close symbiosis with tropical sea anemones, anemonefishes are also sequential hermaphrodites^37^. Typically, anemonefishes form family groups with a size-dependent hierarchy consisting of several smaller sexually immature individuals and a dominant sexually mature pair out of which the largest individual is the female. Due to their striking appearance and lifestyle, we were interested to know how their visual system might be adapted to their visual ecology and behaviours.

We focused our study on the Barrier Reef Anemonefish, *Amphiprion akindynos*, a species mainly found on the Great Barrier Reef in Australia. Using high-throughput RNA sequencing (RNAseq), we first resolved opsin gene expression and tested whether gene expression varies across sex and/or the size of fishes. We then developed a fluorescent *in situ* hybridization method (FISH) to ascertain opsin specificity to a photoreceptor type, either single or double cones, identify potential opsin coexpression, and assess intraretinal variability in expression patterns. Furthermore, we determined the spectral absorbance of visual pigments by a combination of direct photoreceptor absorbance measurements and estimates. We then combined intraretinal patterns in opsin gene expression with the visual pigments‘ spectral absorbance, and with photoreceptor and ganglion cell topographic maps. This allowed us to identify the different areas of their visual field and their potential functional differences and relate these to their ecology and behaviour. Finally, using the newly constructed visual system, we modelled how the visual capabilities of *A*. *akindynos* are linked to primary visual tasks such as feeding, and the detection of their host anemone, conspecifics and predators.

## Results

### Opsin gene repertoire and gene expression

**Figure 1:**
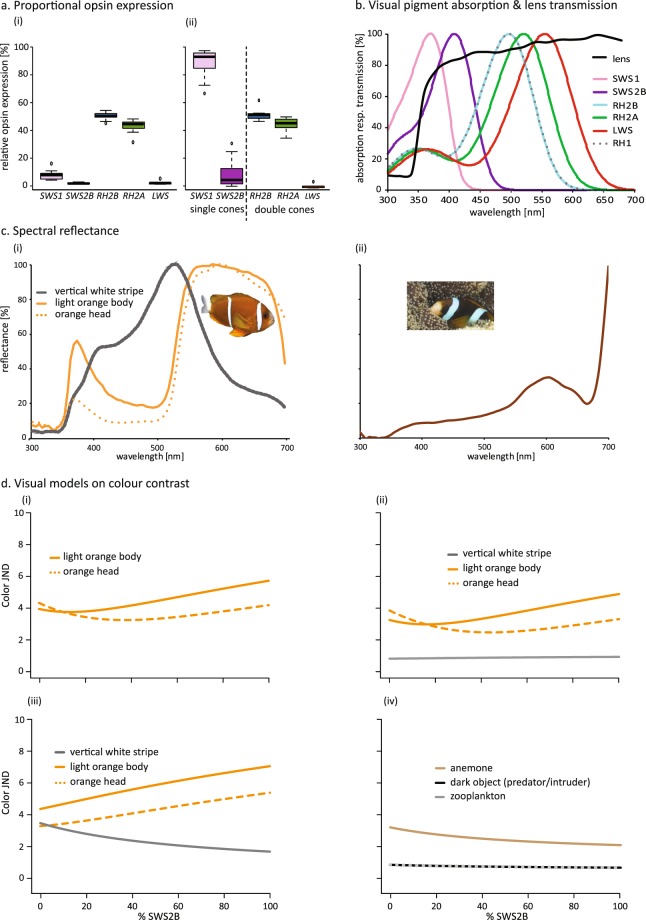
Relative opsin gene expression (**a**), visual pigment λ_max_ and lens transmission (**b**), body and host anemone spectral reflectance (**c**), and colour contrast modelling (**d**) in *Amphiprion akindynos*. (**a**) Relative expression of cone opsin genes as a fraction of all cones (i), and as a fraction of single (*SWS1* and *SWS2B*) versus double (*RH2B*, *RH2A*, and *LWS*) cones (ii). No difference in opsin gene expression between sex or size were found (Table S2). Hence, plots represent data for all specimens combined (total n = 10; 4 females, 2 males, 4 immatures). The box indicates Q2 and Q3, with the line indicating the median. The whiskers indicate Q1 and Q4 of the data, with dots marking outliers. (**c**) Idealised spectral absorbance curves for cone visual pigments matched to opsin proteins. λ_max_ for RH1, RH2B, and RH2A was measured using microspectrophotometry, whereas λ_max_ for SWS1, SWS2B and LWS was estimated based on protein structure. Light transmission curve (black line) of the lens showing UV-transmittance (example of a female lens). (**c**) Normalised spectral reflectance measurements of different body parts of *A*. *akindynos* (i), and one of its host anemones (ii). (**d**) Colour contrast calculated for distinguishing pairs of targets (indicated as just noticable differences, JND) as % SWS2B expression is increased in single cones also expressing SWS1: head orange (450 nm) or body orange (447 nm) versus white stripe (i), fish colours [head orange (446 nm), body orange (444 nm) and white stripe (370 nm)] versus horizontal radiance (ii) fish colours [head orange (449 nm), body orange (447 nm) and white stripe (464 nm)] versus anemone (iii) other targets [anemone (370 nm), dark predator (520 nm) and zooplankton (370 nm)] compared to the horizontal radiance (iv). Number in parentheses indicate the best visual pigment for distinguishing that colour combination based on the peak sensitivity of a visual pigment with a certain %SWS2B.

In total, retinal transcriptomes from ten *A*. *akindynos* individuals (four females, two males, four immature) were sequenced. We confirmed the repertoire of opsin genes known for *A*. *akindynos*^38^, and were able to obtain the complete coding regions of *SWS1*, *SWS2B*, *RH2B*, *RH2A*, *LWS*, and *RH1* (for opsin accession numbers, see Table S1). Proportional expression as a fraction of the total of all opsin genes expressed was 50.3 ± 12.0 (mean and standard deviation) for rods and 49.7 ± 12.0 for cones. Proportional expression as a fraction of total cone opsin expression was for: *SWS1 = *6.8 ± 3.6%, *SWS2B* = 0.7 ± 0.5%, *RH2B* = 49.2 ± 3.0%, *RH2A* = 42.0 ± 5.0%, and *LWS* = 1.3 ± 1.5% (Fig. 1ai). Proportional expression as a fraction of single and double cone gene expression calculated separately, resulted in single cone expression of *SWS1* = 89.3 ± 10.7% and *SWS2B* = 10.7± 10.7%, and double cone expression of *RH2B* = 52.8 ± 4.1%, *RH2A* = 45.9 ± 4.6%, and *LWS* = 1.4 ± 1.7% (Fig. 1aii). No significant correlations between expression patterns and sex or size of individuals were found (Table S2).

### Spectral sensitivity

We used two different methods to gather visual pigment spectral sensitivities; measurements of opsin protein absorbance (MSP), and estimates based on amino acid sequences (Fig. 1b, Table S3). Mean λ_max_ of different photoreceptor types found in *A*. *akindynos* (MSP-based; n fish = 3, being kept in aquaria for 4 months (n = 1), or 6 months (n = 2)) were matched with opsin genes within the damselfish family^28,31^, and are listed in Table S3. Exemplary absorption curves for each visual pigment are shown in Fig. S1. A summary of estimated λ_max_ values is given in Table S3. The aminoacids that were considered for this approach and estimated substitution effects thereof are listed in Table S4.

In theory, λ_max_ values should match between approaches. This was the case for the rods, where MSP and estimates revealed a λ_max_ of 498 ± 4 nm (n = 23) and 496 nm, respectively. Also, for medium-wavelength-sensitive double cone members, MSP revealed a ‘green’ visual pigment with 520 ± 5 nm (n = 13) λ_max_ which matched the estimations for RH2A (516/518/523 nm λ_max_), and a ‘blue’ visual pigment with 498 ± 4 nm (n = 12) λ_max_ which matched one estimate for RH2B (498 nm λ_max_). It is worth noting that RH2B estimates varied depending on the reference species used (Table S4). Since those differences are not fully explained by substitutions in known tuning sites, additional undescribed tuning sites or more general differences in the protein may cause λ_max_ shifts in damselfishes. MSP and estimates differed for the long-wavelength-sensitive double cone member and for the single cones. While MSP λ_max_ for the ‘red’ visual pigment was 541 nm (n = 1), the LWS λ_max_ estimate was 554 nm. However, MSP might not be reliable as it was only based on one cell due to the low *LWS* expression (Fig. 1a), and showed a lot of deviation from the visual pigment nomogram. Furthermore, single cone λ_max_ by MSP was 400 ± 3 nm (n = 6), but λ_max_ estimates were 370 nm for SWS1 (‘UV’) and 408 nm for SWS2B (‘violet’).

### Lens transmission

As lenses have been shown to be the primary light-filter of the damselfish eye^4^, we measured lens transmittance [defined by the wavelength of 50% of maximal transmittance (T50)] in 8 individuals (3 females, 2 males, 3 immatures), and found that the lenses were UV-transmitting independent of ontogenetic stage. T50 was 358 ± 3 nm for females (Fig. 1b), 352 ± 9 nm for males, and 328 ± 8 nm for immature specimens.

### Spectral reflectance

We gathered spectral reflectance of one species of host anemone, and three living individuals of *A*. *akindynos* of different life/sex stages. No differences in reflectance spectra were observed between life/sex stages. Figure 1c illustrates spectral reflectance data for *A*. *akindynos* (i), and one of its host anemone (ii). White stripes reflect from <400 nm (UV) to beyond 700 nm (far-red, not shown in figure) with a peak around 520 nm, while oranges show a small peak in the UV (360–380 nm) and another larger one at longer wavelengths (500–700 nm). The anemone reflects from the UV to the far-red (not shown in figure) with peaks around 600 nm.

### Visual modelling

We performed visual modelling to explore how the different visual pigments might contribute to a fish’s ecology including conspecific, prey, and predator detection. Damselfishes are potentially trichromatic in colour sense but we first considered the optimal monochromatic visual pigment for detecting contrasts as this helps delineate which pigments are best for each visual task. As results were insensitive to which lens was used (that for a male, female or immature), we used the male lens for all subsequent results. As this study proves that single cone expression is very specific for *A*. *akindynos*, i.e., coexpression is only found in a localised area, we then modelled how coexpression in the single cones could affect a typical trichromatic *A akindynos* visual system.

The best monochromatic pigment varied with target combinations (Fig. S2). For the majority of *A akindynos* colours versus each other (Fig. S2a), versus the host anemone (Fig. S2b), or versus the horizontal spacelight (Fig. S2c), the best visual pigment is in the 444–450 nm range. This is the location where the colours of the white stripe and the orange patches were most different from each other and from the horizontal radiance and anemone backgrounds. The one exception for fish colours was the contrast for the white stripe against the side welling radiance, that was better for a very short pigment (370 nm). Non-fish colours also showed specific optima (Fig. S2d). The anemone was best distinguished from the horizontal radiance at 370 nm, and so was zooplankton, although the wavelength dependence for the latter was minimal. Similarly, the wavelength dependence of detecting a dark looming predator was relatively flat, though slightly improved at a longer wavelength of 520 nm. This would make it a matching pigment to the average horizontal radiance.

For the trichromatic *A. akindynos*, we compared the colour contrast of different target combinations while varying how much SWS2B was coexpressed with SWS1 in the single cones (Fig. 1d). We found that the majority of fish colours (*A*. *akindynos*) against each other (Fig. 1di), or against the anemone (Fig. 1dii) or background spacelight (Fig. 1diii), improved for higher SWS2B expression. This was particularly true for the body orange colour against white stripe. In addition, the head orange against the anemone or white stripe was best distinguished when having either very low or high SWS2B coexpression.

For detecting the white stripe (Fig. 1diii) or anemone (Fig. 1div) versus horizontal radiance, higher SWS1 expression was found to be best. For zooplankton and predator detection against the spacelight, those were relatively insensitive to single cone coexpression (Fig. 1div).

### Cone photoreceptor arrangement

**Figure 2:**
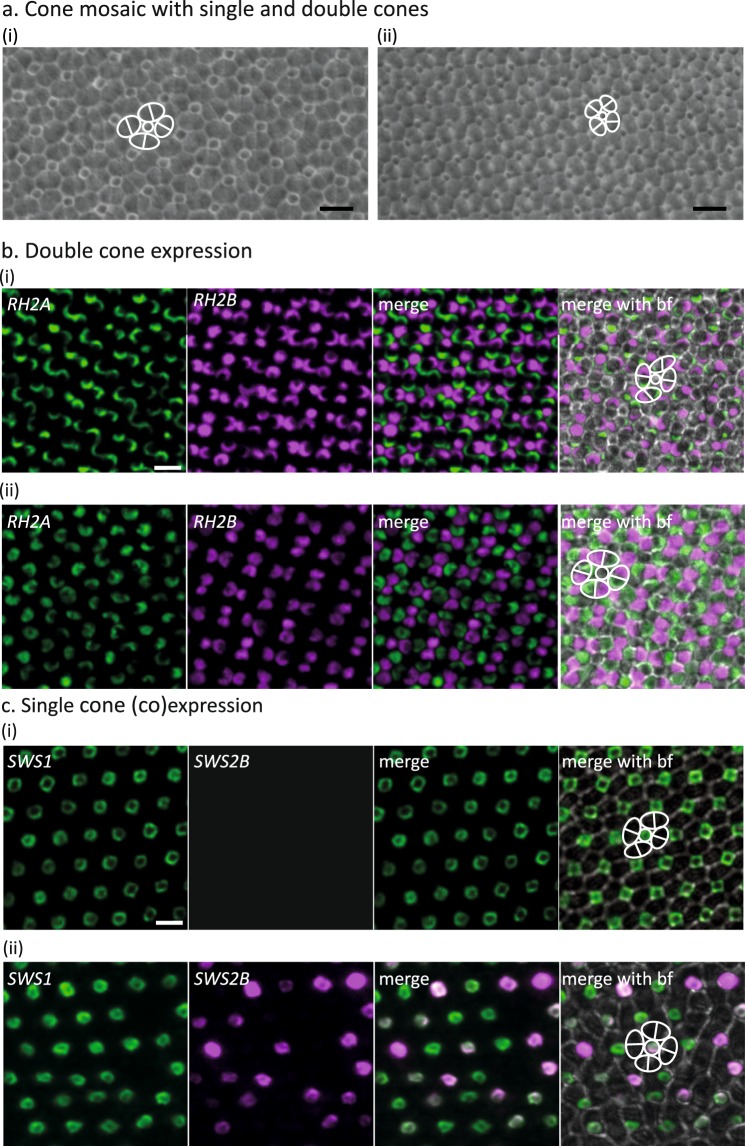
Cone mosaic (**a**) and double (**b**) and single (**c**) cone opsin expression revealed by fluorescent *in situ* hybridization in *Amphiprion akindynos*. (**a**) Cone mosaic in the nasal (i) and temporal (ii) areas of the retina (note that cone densities are higher in the temporal area). Every single cone (white circle) is surrounded by four double cones (white ovals), each composed of two members (separated by white lines). (**b**) High resolution images show that *RH2A* (green) and *RH2B* (magenta) are expressed in opposite members of every double cone throughout the retina [(i) nasal, (ii) temporal area of retina according to inserts of whole retina scans seen in Fig. S3a]. (**c**) High resolution images show that *SWS1* is expressed in every single cone throughout the retina [(i) nasal area of retina], whereas *SWS2B* is only expressed in some single cones forming a small area located in the temporal area (ii) (for whole retina scans according to inserts (i, ii), see Fig. S3b). Importantly, *SWS2B* is always coexpressed with *SWS1* (ii). Brightfield (bf) images in (**b**,**c**) show the cone mosaic. Scale bars: (**a**–**c**) 10 µm.

Cones in *A*. *akindynos* were arranged in a regular fashion forming a mosaic composed of single cones each surrounded by four double cones (Fig. 2a), with a double to single cone ratio of 2:1.

### Fluorescent *in situ* hybridization (FISH) of opsin genes

FISH on retinas of *A*. *akindynos* illustrated that the longer wavelength-sensitive genes, *RH2A*, *RH2B* and *LWS*, were expressed in double cones only. *RH2A* was expressed in one member and *RH2B* in the other member in >99% of double cones (Figs 2b and S3a). *LWS* was only detected in very few cells (<1%; Figs S4 and S5).

The short-wavelength-sensitive genes, *SWS1* and *SWS2B*, were expressed in single cones only (Figs 2c and S3b). While all single cones expressed *SWS1* (Fig. 2ci), *SWS2B* was found to be coexpressed with *SWS1* in very few cells (Fig. 2cii). Moreover, the coexpression of *SWS* genes was restricted to a specific small region in the temporal part of the retina as shown by whole retina scans (Fig. S3bii).

### Topographic distribution of ganglion cells, cone photoreceptors, and opsin genes

**Figure 3:**
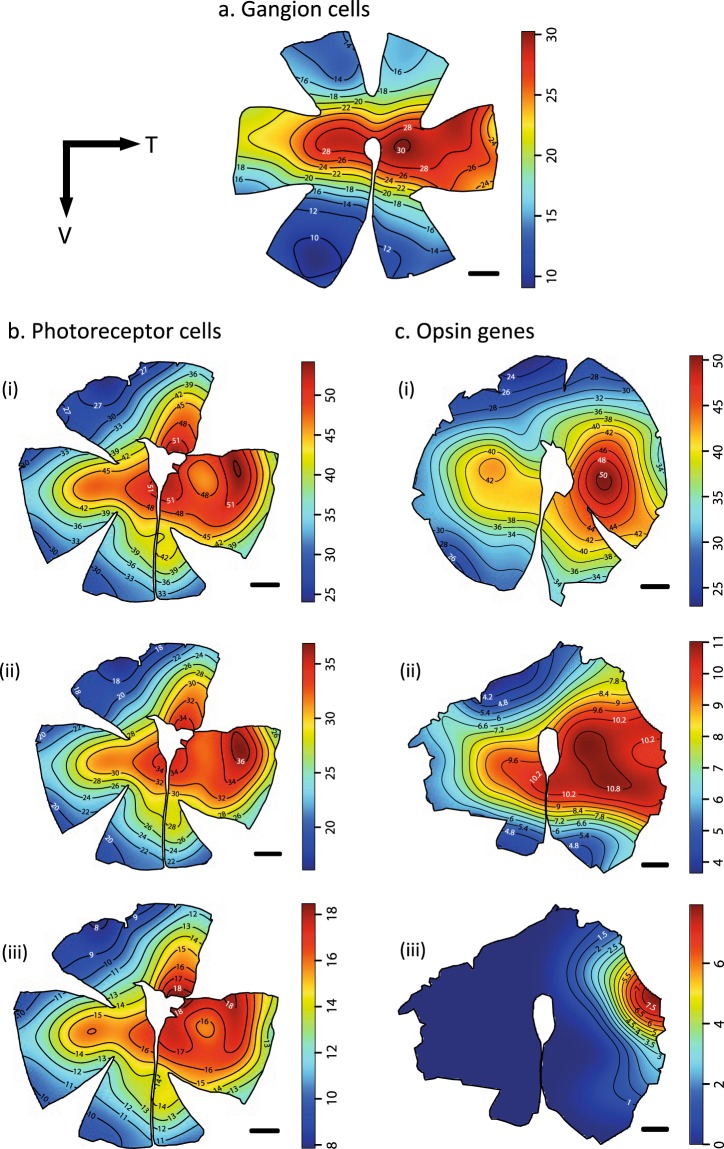
Topographic distribution of the different neural cells in the retina of *Amphiprion* *akindynos*. (**a**) Retinal ganglion cells, (**b**) photoreceptor cells with (i) total cones, (ii) double cones, (iii) single cones, and (**c**) FISH-based opsin gene expression shown for (i) *RH2s* (*RH2A* and *RH2B*), (ii) *SWS1*, (iii) *SWS2B*. The black lines represent iso-density contours and values are expressed in densities × 10^3^ cells/mm^2^. The black arrow indicates the orientation of the retinas. T = temporal, V = ventral. Scale bars: 1 mm.

For the retinal topography analyses, the eyes of several individuals were analysed: seven for photoreceptor analyses (3 females, 2 males and 2 immature), three for ganglion cell analyses (1 female, 1 male and 1 immature), and eleven for *in situ* analyses (4 females, 3 males, 4 immature). The topographic distribution of ganglion cells, cone photoreceptors, and opsin genes did not differ between female, male and subadult individuals. As such, only results for one female are presented here (Fig. 3), with the rest of the results available in the Supplementary Figs S5–S10.

#### Ganglion cell densities

There were only few differences in the ganglion cell topographies between individuals at different ontogenetic stages (Fig. S6). All three individuals analysed possessed a horizontal streak (i.e., elongated increase in cell density along the retinal meridian) with a peak density of cells in the temporo-central part of the retina close to the optic nerve (Fig. 3a). The horizontal streak was more pronounced in the temporal part of the retina than elsewhere. The total number of ganglion cells varied greatly with the size of the fish, from 432,756 cells in the immature individual (4.1 cm total length (TL)) to 969,964 cells for the female (8.8 cm TL; Table 1). The peak ganglion cell densities were similar between individuals ranging from 40,400 cell/mm^2^ in the female to 45,000 cell/mm^2^ in the immature individual. Consequently, the spatial resolving power (SRP) varied greatly between ontogenetic stages, becoming higher as the fish gets bigger and ranging from 3.63 to 5.80 cycles/deg in immature and female fish, respectively (Table 1). Using these SRP values, a female or an immature fish should be able to distinguish another individual that measures 8 cm TL from up to 26 m and 16 m away, respectively. They should also be able to distinguish a single white stripe, 5 mm in width, that corresponds to the size of a white stripe on a 8 cm TL fish, from 1.6 m and 1 m away, respectively.

**Table 1:** Summary of the ganglion cell quantitative data.

Sex	Indiv.	Total length (cm)	Total number	Peak density (cells mm^−2^)	CE	Lens ∅ (mm)	SRP (cycles deg^−1^)
Female	1	8.8	969964	40400	0.030	2.5	5.77
Male	1	8.4	964867	44000	0.032	2.4	5.80
Immature	1	4.1	432756	45200	0.032	1.4	3.63

Data is obtained using the optical fractionator methods on retinal wholemounts of *A*. *akindynos* of different sex. ∅ = diameter, SRP = spatial resolving power.

#### Cone densities

The regular cone mosaic pattern of one single cone surrounded by four double cones (Fig. 2a) was consistent over the entire retina, resulting in similar topographic distributions for each cell type. As a result, we only provide and describe in detail the distribution pattern for total cones. The topographic distribution of total cones varied slightly between individuals irrespective of their size and sex (Fig. S7). However, the overall topography did not differ between fishes. Similar to the ganglion cell distribution, cone densities formed a horizontal streak with a peak density in the temporal or temporo-central part of the retina. However, this streak was generally wider for cones than ganglion cells, extending vertically into the temporal part of the retina (Fig. S7). This resulted, for some individuals, in a very wide area of increased cone cells densities in the temporal part of the retina (Figs 3bi, S7b,d). Other individuals possessed a less pronounced but more defined vertical component in the temporal part of the retina as well as two areas (i.e., concentric increases in cell densities) in the nasal and temporal part of the retina within the horizontal streak (Fig. S7e,f).

Total cone numbers varied from 667,466 for the smallest individual (3.2 cm TL) to 1,884,932 for one of the larger individuals (7.3 cm TL; Table 2). The peak total cone densities varied from 61,200–89,200 cell/mm^2^, with an average of 76,286 cells/mm^2^ (Table 2). However, since no subsampling was performed for photoreceptor counts, these values may not necessarily represent the highest cone density for each individual. For one individual (immature #2; Tables 1 and 2), both ganglion cells and photoreceptors were mapped (Figs S6c and S7f, respectively). For this individual the ratio of cone to ganglion cells varied from 1.5:1 in the peak ganglion cell area to 3:1 in the ventral part of the retina, with an average of 2:1 across the retina.

**Table 2:** Summary of the photoreceptor cell quantitative data.

Sex	Indiv.	TL (cm)	Total SC	Peak SC (cells mm^−2^)	Total DC	Peak DC (cells mm^−2^)	Total cones	Peak TC (cells mm^−2^)	CE
F	1	4.6	341231	29600	683617	60400	1024848	89200	0.033
	2	7.3	637762	28800	1247170	56000	1884932	83200	0.032
	3	8.9	630170	20400	1213522	42000	1843692	61200	0.033
M	1	6.5	617061	24400	1248688	49200	1865749	73600	0.034
	2	7.8	603371	23200	1177989	46400	1781360	69600	0.033
I	1	3.2	223220	25200	444245	50800	667466	76000	0.037
	2	4.1	297794	27200	592321	54000	890116	81200	0.030

Data is obtained using the optical fractionator method on retinal wholemounts of *A*. *akindynos* of different sex. F = female, M = male, I = immature, TL = total length, SC = single cones, DC = double cones, TC = total cones.

#### Opsin gene densities

The topographic distribution of cone photoreceptors expressing specific opsin genes was analysed using FISH on whole mounted retinas. Since *SWS1* was found to be expressed in all the single cones and *RH2A*/*RH2B* in the vast majority of double cones, we expected to find similar topographies and densities as for single cone and double cone topographic maps. While this was more or less the case for some individuals (Fig. 3), topographic maps from FISH data were in general less reliable, showing greater variation in topography between individuals and underestimating densities (Figs S6 and S7). This can be explained by the more intrusive methods used during FISH resulting in the loss of several photoreceptor cells. An example of such an under-labelled retinal region is shown in Fig. S8c.

Nevertheless, FISH-based topographic maps were very useful to identify the peculiar distribution of the cones expressing *SWS2B*, which were found to be coexpressed with *SWS1* in a small area of the temporal part of the retina. *SWS2B* cone densities formed an area centralis with a peak density of cells toward the retinal margin of the temporal area (Fig. 3ciii). This specific distribution was consistent between the three individuals irrespective of sex and size (Fig. S10). This small area centralis at the temporal margin of the retina was therefore the only area in the retina that coexpressed two opsin genes. This area also showed a high-density of ganglion cells, although lower than the area of highest ganglion cell density located toward the centre of the retina (Fig. 3a,ciii).

Topographic maps of *LWS* cone densities could not be created due to the extremely low number of cells expressing this gene (<0.1% of all double cones). However, we were able to highlight labelled *LWS* cones across the retina (Fig. S5), which revealed that they are randomly distributed throughout the retina.

## Discussion

The *A*. *akindynos* cone opsin expression (*SWS1*, *SWS2B*, *RH2B*, *RH2A*, and *LWS*, Fig. 1a) was similar to that known in other damselfishes^31^ and appears generally well-suited to its ecology. As such, the prominently expressed mid-wavelength-sensitive *RH2B* and *RH2A* genes, are matched to the environmental spacelight on coral reefs^39^. As for other damselfishes^4,31^, *A*. *akindynos* had UV-transmitting lenses and expressed the UV-sensitive *SWS1* in its single cones. In these fishes, UV vision (Fig. 1a,b) together with UV-reflective body parts (Fig. 1c) indicate the use of a close-range ‘private’ communication channel, likely hidden from ‘UV-blind’ predators^35,40^. Although rare, *SWS2B* expression had previously been reported in some damselfishes^31^. However, to our knowledge, this is the first time *SWS2B* has been shown to be coexpressed with *SWS1* in a coral reef fish. As discussed in detail below, the localised coexpression of these two genes (Fig. 3ciii) may improve the detection of host anemones and conspecifics. Finally, both retinal transcriptomes (i.e., low expression; Fig. 1a) and topographic maps (i.e., few cells, no pattern; Fig. S5) suggest a negligent or even non-functional role of the long-wavelength-sensitive *LWS* opsin in *A*. *akindynos*.

Beyond the ecological significance, the multi-facetted approach of this study highlights how using opsin gene expression alone, without knowing in which cells and where across the retina genes are expressed, may lead to misinterpretations about the visual system of an animal. For example, if looking at expression data only, *SWS2B* may appear of minor importance for anemonefish vision as it was only found to be expressed at very low levels (1% of total cones [Fig. 1ai] and 10% of single cones [Fig. 1aii]). However, by labelling *SWS2B* expressing cells and imaging them across the retina, we were able to show that it is coexpressed with *SWS1* (Fig. 2c) in a restricted region (Figs 3ciii, S3b). In this temporal region of coexpression, the percentage of single cones expressing *SWS2B* goes up to 30–70%, which highlights that a low overall expression level may not necessarily equate to no functionality.

In accordance with findings from other fishes^26,29,41^, our data further suggests that changes in the light habitat may drive plasticity in opsin gene expression in *A*. *akindynos*. MSP revealed a consistent λ_max_ of ~ 400 nm for single cones in fishes that were kept under laboratory conditions for an extended period of time. This spectral sensitivity is longer-shifted compared to the estimated 370 nm λ_max_ for the UV-sensitive SWS1 (measured at 347–376 nm λ_max_ in other damselfishes^15^), but is shorter than the estimated 408 nm λ_max_ for the violet-sensitive SWS2B (Table S3). This suggests that single cones in fishes kept under artificial lighting coexpressed *SWS2B* and *SWS1* across the retina. Since under natural lighting conditions coexpression was restricted to a small area (Figs 2ciii, S3b), a substantial change in gene expression likely occurred when transferring animals to the laboratory. Luehrmann *et al*.^29^ found that in two related damselfish species, *SWS1* expression decreased while *SWS2B* expression increased within a few weeks when moving fish to an artificial, UV-deprived light environment. Our findings, along with others^42^, emphasise the need to take the environment and especially the light habitat into account when drawing conclusions about the visual ecology of an animal. They also show that opsin gene expression and by extension spectral sensitivities only represent the state of a specimen at the time of sampling, a state that may change over time or spatial distribution of the fish (e.g., ontogeny^43^, time of day^44^, season^45^, or depth^28,46^).

The retinal topography of teleost fishes, and especially their ganglion cell topography, closely reflects the ecological characteristics of species^16–18,47,48^. In reef fishes, a clear link between the type of retinal specialization and the symmetry of the visual environment or habitat has been established. While species living in an open environment with an uninterrupted view of a horizon, like the sand to water interface, usually have streaks^17^, species living in a more enclosed, 3D environment with an interrupted horizon, have one or multiple areae of high cell densities^47^. Anemonefishes live in close association with their anemone host and as such, have relatively enclosed and restricted habitat ranges where area specialisations might be expected. Contrary to this expectation, *A*. *akindynos* possess a well-defined horizontal streak with a peak density in the centro-temporal part of the retina (Fig. 3a). A horizontal streak allows fishes to concentrate on a broad range of the environment without distinctive eye and head movements, and may provide a better threshold for movement detection^17^. While this type of specialisation may not fit perfectly with the symmetry of the anemonefish’s habitat, it may be very useful in light of their behaviour and peculiar social hierarchy.

Anemonefishes are highly territorial fishes and will show aggressive behaviour toward any inter- or intraspecific intruders^49,50^. A horizontal streak may therefore allow them to visualise their environment at a long-range and look for possible intruders while staying within the safety of the anemone. The peak-cell density in the central part of the retina provides higher acuity in the monocular field of view screening toward either side of the fish. This may assist during aggressive encounters at a close-range with conspecifics. Anemonefishes use body size to gauge the social status of conspecifics^51^ and this may be assessed through side-by-side swimming at the beginning of an encounter^49^.

Less is known about the retinal topography of photoreceptors in teleost fishes, but it generally matches the pattern seen in their ganglion cells^33,52^. Discrepancies between photoreceptor and ganglion cell topographies may indicate different visual requirements in different parts of the visual system. In addition to the horizontal streak, the photoreceptor pattern of *A*. *akindynos* shows a vertical component in the temporal part of the retina (Fig. 3b), therefore providing increased sensitivity in the field of view situated above and below the fish. This vertical component could be associated with the visualization of vertical body stripes used for species recognition^53^, or help with the detection of predators/intruders situated above or below them.

Adult *A*. *akindynos* have an acuity (SRP) of 5.8 cycles per degree which is relatively low compared to other coral reef fishes (3–27 SRP) and is the lowest recorded so far for any damselfish species^10^. Anemonefishes live in relatively small territories close to their host anemone and interact over a close-range with conspecifics and intruders. As a result, none of their behavioural tasks appear to require high acuity. Spatial resolving power estimation and behavioural observation show that their acuity is high enough to recognise or at least detect conspecifics, even at the level of a single white stripe, from few meters away. Interestingly, immature specimens that occupy smaller territories compared to adults^50^, also have a lower acuity, which restricts the distance at which they can spot a conspecific.

Opsin gene distribution in double cones across the retina was found to show a pattern of *RH2B* expression in one member and *RH2A* in the other. Consequently, their distribution matches the photoreceptor topography i.e., a horizontal streak with a peak density in the centro-temporal part and a vertical component in the temporal part of the retina (Fig. 3ci). The double cone photopigments are well matched to the environmental spacelight on coral reefs (i.e., from blue to green^39^), which may help detect intruders against the background. Indeed, visual models show that the detection of looming dark objects (e.g., predators or competitors) is best achieved at longer wavelengths, similar to the λ_max_ of the RH2 photopigments. Interestingly, cichlids also tune their double cone sensitivities to the prevalent light environment presumably to increase the detection of prey, predators, or mates^32^.

Topographic analysis of single cone opsin densities across the retina highlighted: (1) *SWS1* is present in every single cone and therefore has the same topography as the photoreceptors and the double cone opsins (Fig. 3cii); and (2) a novel specialisation in fishes, in the form of an area temporalis formed by the coexpression of *SWS2B* with *SWS1* (Fig. 3ciii). Our monochromatic modelling showed that UV-sensitivity, as provided by the pure expression of an SWS1-based visual pigment, helps, albeit not in an outstanding manner, in luminance detection of zooplankton (Fig. S2d). This agrees with previous findings looking at the function of UV-sensitivity in fish foraging^54,54^. Furthermore, the mono- and trichromatic models also revealed that the use of SWS1-based single cones increases the colour contrast of the host anemone against spacelight (Figs 1div and S2d). The colour contrast of conspecifics, however, varied depending on the degree of SWS2B coexpression and the patterns that were compared (Fig. 1di–iii).

While this is the first report of *SWS2B* and *SWS1* being coexpressed in a coral reef fish, their orthologs have previously been found to be coexpressed in the single cones of the cichlid *Maylandia zebra*^33^. However, both the distribution and function of the coexpression seem to differ between species. In *M*. *zebra*, coexpression showed a very high intraspecific variability both in topography and level of gene expression. On the other hand, in *A*. *akindynos*, all individuals showed a similar topographic pattern irrespective of sex/life-stage, indicating that the coexpression has a defined purpose in this species. Furthermore, in the cichlid opsin gene coexpression was prevalent throughout the retina except for the central part of the retina representing an area of high ganglion cell density. In the anemonefish, by contrast, the coexpression was restricted to the temporal part of the retina to an area of high cell density (Fig. 3ciii). Finally, visual modelling showed that this coexpression is likely to increase achromatic contrast detection in the peripheral visual system of *M*. *zebra*^33^. In *A*. *akindynos*, on the other hand, coexpression may improve the chromatic contrast of orange body colour versus white stripes of a conspecific situated in front of the fish as well as orange body colour versus the anemone host and the spacelight (Fig. 1di–iii). In this case the trichromatic models are similar to the monochromatic ones in that having a visual pigment close to 450 nm improves contrast between fish colours and other targets. Since the single cone visual sensitivity is closest to this wavelength, it is the one best able to improve contrast. Higher *SWS2B* expression enables the single cone to get closer to the 440–450 nm optimum. This specialised area of coexpression might then have evolved to better recognize conspecifics to maintain hierarchy and avoid territorial bouts. It might also have evolved to readily recognize other anemonefish species when sharing a host, thus generally reducing aggression between species. Detailed investigations of the visual systems/ecology of related damselfish species and of anemonefishes in particular, as well as behavioural experiments will help answer these questions in the future.

## Methods

### Specimen collection

All *A*. *akindynos* specimens were either collected on reefs surrounding Lizard Island, Australia, or obtained from an aquarium supplier (Cairns Marine) collecting fishes from the Northern Great Barrier Reef. Fish were anaesthetized with Tricaine methanesulfonate (Sigma-Aldrich) or an overdose of clove oil, and killed by decapitation. Specimens were classified as subdominant males, dominant males (as defined by the presence of testes and/or being the largest male of the group), and females (as defined by the presence of ovaries and/or being largest individual of the group), and size (total length) as well as affiliation to the same family (as defined as specimens living in the same anemone) were noted. A summary of the number of individuals used with their sex, size and family, where they were sourced from, and for which type of analysis they were used is provided in Table S5. A detailed description of the methods used in this study is provided in the Supplementary Information and move the whole sentence and place it directly under Methods (before Specimen Collection).

### Opsin gene study

#### Transcriptomic sequencing and processing

For each individual, the retina from one eye was dissected out and preserved in RNAlater (Ambion) until further processing. Transcriptomes were sequenced on an Illumina HiSeq 2000 and processed following previously published methods^21,55^. Trinity was used for *de-novo* assembly of transcripts that were mapped to known and publicly available opsin genes of *A*. *akindynos* (HQ286499, HQ286509, HQ286519, HQ286529, HQ286539, HQ286549). Further bioinformatics analyses were performed using Geneious software (Version 9.0.4).

#### Opsin gene expression

We analysed proportional cone and rod opsin expression as a fraction of the total of all opsin genes expressed. To test for cone expression and as this study shows evidence that damselfish single cones only express the *SWS* opsin genes whereas double cones express *RH2* and *LWS* opsin genes, we analysed the relative expression of opsin genes as a fraction of either total cone expression or as single and double cone opsin expression separately.

In order to test whether sexual status or size had an effect on cone opsin gene expression, we used a beta regression as implemented in the R package BETAREG^56^, which allows handling of non-transformed data to model percentages and proportions. Statistical analyses were performed in R^57^ using the interface RSTUDIO v.0.98.1062.

### Visual pigments maximal absorbance (λ_max_)

#### Microspectrophotometry (MSP)

Operation of MSP followed a standard protocol developed for vertebrate and invertebrate photoreceptors from e.g., Hart^58^, and raw absorbance spectra were analysed as described in Hart *et al*.^59^.

#### λ_max_ predictions

Due to the limitations of MSP in gathering information on visual pigments that are sparse across the retina or expressed at low-levels, and to additionally confirm λ_max_ of opsins measured by MSP, we further estimated the λ_max_ of each opsin protein based on differences of their amino acid sequences to reference species. We focused on variable amino acid residues that either occurred in areas corresponding to the retinal binding pocket and were substitutions resulted in a change in polarity, or at known tuning sites.

### Lens transmission

We gathered lens transmission curves (300–800 nm) following previously published protocols^4^. Transmission curves were normalized at 700 nm, and the wavelength at which 50% of the maximal transmittance (T50) was reached was determined using a linear regression^5,60^. We then assessed whether the *A*. *akindynos* eyes would be UV blocking (T50 > 400 nm) or UV transmitting (T50 < 400 nm).

### Spectral reflectance

Spectral reflectance of different areas of the fish with focus on white stripes and orange body as well as different areas of the anemone were measured following the methods described in Marshall *et al*.^61^.

### Visual modelling

Because very little is known about damselfish photoreceptor opponency, modelling was performed using the receptor noise limited model which does not require any explicit understanding of processing post-photoreceptor^62,63^. This enabled us to explore the relative function of various visual pigment combinations in performing different visual tasks. For the monochromatic modelling, we tried all the possible lens transmissions (male, female, and juvenile) to see if they had any impact on the results.

Visual tasks included conspecific, prey, and predator detection. Conspecific detection included comparing *A*. *akindynos* colours against each other or against the spacelight or an anemone. The foraging task was to detect zooplankton against the background spacelight. Since zooplankton scatters downwelling light, and has a somewhat flat reflectance into the UV^64,65^, we modelled zooplankton as having a flat reflectance of 10% and assumed it scattered the higher intensity downwelling irradiance. Detecting a predator was modelled as discriminating a dark grey object against the background spacelight as would occur for a looming stimulus. This was modelled as a dark grey with a flat reflectance of 10%, which was assumed to scatter the horizontal irradiance.

### Quantum catch

We calculated the quantum catch of different photoreceptors as they viewed the light coming from different targets. Fishes or anemone were illuminated by horizontal irradiance, while zooplankton was illuminated by downwelling irradiance. One additional target was the horizontal radiance from a small patch of the background space light. Downwelling irradiance, and horizontal radiance and irradiance were obtained from previous measurements in reefs around Lizard Island^28^.

### Visual discrimination

We use the receptor noise limited model to quantify the relative discrimination of two targets based either on luminance or colour contrast^62,63,66–68^. Luminance contrast is calculated using the receptor noise-based contrast to be consistent with colour discrimination, providing the results in terms of just noticeable differences (JNDs) i.e., the threshold at which two objects should be distinguishable from one another.

As shown in Fig. 2, *A. akindynos* has one single cone for each pair of double cones so that n_S_: n_M_: n_L_ = 1: 2: 2. We further set the Weber fraction, ν_L_ to be 0.1, based on colour experiments in other fishes^69,70^. Luminance noise has not been quantified, so we assume that the Weber fraction is similar for both colour and luminance^68^. In these calculations, we then vary either the peak λ_max_ for a given photoreceptor or the amount of coexpression of the *SWS1* and *SWS2B* opsin genes in the single cones. Since we are making relative comparisons between visual pigments with different peak sensitivities or different coexpression values, the actual Weber fraction does not impact our conclusions.

### Retinal wholemount preparation

#### For photoreceptor and ganglion cell topographies

The lenses and corneas were dissected out and the eye cups fixed in 4% paraformaldehyde (PFA) in 0.1 M phosphate buffer saline (PBS, pH = 7.4) overnight at 4 °C. Retinal wholemounts were then prepared according to standard protocols^71–73^.

#### For *in situ* analyses on wholemount retinas

Eyes were enucleated and prepared following published methods^74^.

### Fluorescent *in situ* hybridization (FISH)

#### FISH protocol

We performed dual-labelling FISH following previously described methods^32,75,76^ with the difference that we reversed transcribed RNA using the High Capacity RNA-to-cDNA kit (Applied Biosystems), and subsequently used cDNA to generate probe templates by standard PCR using the MyTaq^TM^ HSRED DNA Polymerase (Bioline) with primers (listed in Table S6) designed to target the 3′ untranslated region (3′UTR) (*RH2B* and *RH2A*) or the coding sequence (*SWS1*, *SWS2B*, and *LWS*).

#### Image acquisition of labelled opsins

For visualization of the distribution of labelled opsin genes throughout the retina, one exemplary whole retina scan for each dual-labelled opsin pair was performed using a spinning-disk confocal microscope (Spectral Applied Research).

### Stereological analysis and topographic map construction

For each analysis, the outline of the retina was digitized using a x5 objective (numerical aperture 0.16) mounted on a compound microscope (Zeiss Imager.Z2) equipped with a motorized stage (MAC 6000 System, Microbrightfield, USA), a digital colour camera (Microbrightfield, USA) and a computer running Stereo Investigator software (Microbrightfield, USA).

#### Photoreceptor and ganglion cell densities

Following the protocols described in de Busserolles *et al*.^17,77^, topographic distribution of single cones, double cones, total cones and ganglion cells were assessed using the optical fractionator technique^78^ modified by Coimbra *et al*.^79,80^ for use in retinal whole mounts.

Topographic maps were constructed using the statistical program R v3.5.0 (R Foundation for Statistical Computing, 2018) with the results exported from Stereo Investigator software according to Garza-Gisholt *et al*.^81^. For each map we used the Gaussian Kernel Smoother from the Spatstat package^82^ and adjusted the sigma value to the grid size.

#### Opsin gene densities

For each wholemount, around 200 image stacks were randomly and systematically acquired using the *SRS image stack flow function* from the Stereo Investigator software. After additional processing using custom ImageJ scripts to remove background and enhance labelled photoreceptor cells, cells were counted for each colour channel in each counting frame.

Finally, topographic maps of opsin densities were constructed using R and a custom script adapted from Garza-Gisholt *et al*.^81^.

### Spatial revolving power estimation

The upper limit of the spatial resolving power (SRP) in cycles degrees i.e., visual acuity, was estimated for each individual using the ganglion cell peak density as described by Collin & Pettigrew^10^.

### Ethics approval and consent to participate

*A*. *akindynos* specimens were collected under the Great Barrier Reef Marine Park Permit G12/35005.1 and the Queensland General Fisheries Permit 140763. All experimental procedures were approved by The University of Queensland Animal Ethics Committee (QBI/223/10/ARC/US AIRFORCE (NF) and QBI/192/13/ARC), and all experiments were performed in accordance with relevant guidelines and regulations.

## Supplementary information

supplementary information: A detailed investigation of the visual system and visual ecology of the Barrier Reef anemonefish, Amphiprion akindynos

## Data Availability

Opsin sequences and transcriptomic data of this study have been deposited in GenBank (BioProject ID: PRJNA547682), and accession numbers are listed in Table S1. Primer sequences used are given in Table S6.
