# Supplementary material for: A detailed investigation of the visual system and visual ecology of the Barrier Reef anemonefish, Amphiprion akindynos
Source: Sci Rep. 2019 Nov 11;9:16459. doi: 10.1038/s41598-019-52297-0 (PMC6848076; doi:10.1038/s41598-019-52297-0)
Supplement: Supplementary file 1 — supplementary information: A detailed investigation of the visual system and visual ecology of the Barrier Reef anemonefish, Amphiprion akindynos [file 41598_2019_52297_MOESM1_ESM.pdf]

**Supplementary Information**

# A detailed investigation of the visual system and visual ecology of the Barrier Reef anemonefish, *Amphiprion akindynos*

Sara M. Stieb (SMS) [1,2,*,#], Fanny de Busserolles (FdB) [1,*,#], Karen L. Carleton (KLC)[3], Fabio Cortesi (FC)[1], Wen-Sung Chung (WSC)[1], Brian Dalton[3] (BD), Luke A. Hammond[1] (LAH), N. Justin Marshall (JM) [1]

*these authors contributed equally to this work

[1]Queensland Brain Institute, The University of Queensland, Brisbane, QLD 4072, Australia

[2]Centre of Ecology, Evolution and Biogeochemistry, EAWAG Swiss Federal Institute of Aquatic Science and Technology, Kastanienbaum, Switzerland; and Institute of Ecology and Evolution, University of Bern, Switzerland

[3]Department of Biology, University of Maryland, College Park, MD 20742, USA

[#]Corresponding authors: sara.stieb@eawag.ch; f.debusserolles@uq.edu.au

## Material and Methods

### Opsin gene studies

*(a) Transcriptomic sequencing and processing*

Retinas were homogenized using a TissueLyser LT (Qiagen, Netherlands) and total RNA was extracted with the RNeasy Mini Kit (Qiagen, Netherlands) including an optional DNAse digestion step. RNA was quality checked with an Agilent 2100 BioAnalyzer 6000 NanoChip (Agilent Technologies, USA). RNAseq libraries were made using the TruSeq RNA Sample Preparation Kit v.2 (Illumina, San Diego, USA), and transcriptomes were sequenced as 125bp paired-end fragments on an Illumina HiSeq2000 using chemistry v4. Samples were multiplexed at 12 samples per lane obtaining between 1 – 37 million sequenced reads per sample.

Transcriptomes were then processed following previously published methods [1,2] using the online Bioinformatics platform Galaxy v.1.0.4 (Research Computing Centre, The University of Queensland, Australia) [3]. In short, data were converted using FASTQ Groomer, quality checked using FastQC, and trimmed using customized settings in Trimmomatic. Triniti settings were a group pair distance of 250 bp, and minimum inchworm kmer coverage of 2.

*(b) Opsin gene expression*

To analyse differences in opsin gene expression, we mapped the unassembled filtered PE reads against the CDSs of genes extracted from the transcriptomes as per Cortesi et al. [1] and de Busserolles et al. [2].Proportional gene expression was then calculated according to

$$T_i/T_{all} = N_i// \sum N_i \qquad (1)$$

where $T_i/T_{all}$ is the gene expression ratio for a given gene $T_i$ normalized by the total genes expressed in cones and rods, all cones, in all single cones or in all double cones $T_{all}$, and $N_i$ is the number of mapped reads for a given gene divided by its length.

### Visual pigments maximal absorbance ($\lambda_{max}$)

*(a) Microspectrophotometry (MSP)*

Prior to the retinal preparation, fish were dark-adapted for at least 2 hours. Eyes were removed under dim red illumination (690 nm LED). Retinal preparations were conducted under infrared illumination with the aid of a dissecting microscope fitted with an infrared image converter (Electrophysics, USA). The cornea and lens were removed, and the retina was cut into small pieces in 0.1 M phosphate buffer saline (PBS) (17-515DPBS, Lonza, USA). The retinal samples were mounted in 6% sucrose mixed 0.1 M PBS on a coverslip.

Operation of MSP followed a standard protocol [22,23]. The measuring light beam was set to a size of around 2 x 5 µm and placed parallel to the long axis of the outer segment. Baseline and sample scans were made from tissue-free and cellular regions of the preparation respectively. Subsequently, the visual pigments were bleached using a white light beam. Best fit visual pigment nomograms were used to determine the λmax of each sample following former methods [24,25]. Absorbance spectra from each measurement that satisfied the selection criteria [26] were accepted and these data were averaged for each type of photoreceptor.

*(b) $\lambda_{max}$ predictions*

We aligned *A. akindynos* opsin protein sequences with those of reference species [two cichlids (*M. zebra*, and *Oreochromis niloticus*) and the Japanese ricefish (*Oryzias latipes*)], allowing us to identify retinal chromophore binding pocket as well as previously determined tuning sites (i.e. summarized in [27]). Putative spectral sensitivities were based on pure opsin spectral absorbance gained from *in vitro* opsin protein expression studies of *O. niloticus* [28] and/or *M. zebra* [29], depending on which amino acid sequence was closest to the respective *A. akindynos* sequence. As $\lambda_{max}$ estimates of SWS1, RH2A and RH2B were not clear to interpret, we additionally compared the protein sequence of *A. akindynos* to those of other damselfish species (*Pomacentrus amboinensis* for SWS1, *P. amboinensis* and *Dascyllus trimaculatus* for RH2A, and *P. coelestis* and *D. trimaculatus* for RH2B), having a very similar sequence in known tuning sites and with known spectral absorbances gained from MSP [30,31]. We calculated all spectral sensitivities assuming an A1 template as this has been shown to be the most likely retinal pigment found in reef fishes [32,33].

Lens Transmission

Light from a pulsed xenon light source (Ocean Optics, PX2, USA) was directed through the lens mounted above a pinhole and into a quartz fibre-optic cable coupled to a spectrometer (USB2000; Ocean Optics, Dunedin, USA), and five to ten measurements were made per individual.

Spectral Reflectance

The reflectance of different areas of the fish with focus on white stripes and orange body was measured at a 45° angle using a 200 µm bifurcated UV⁄visible optic fibre connected to a PX-2 pulse xenon light source (Ocean Optics) and an Ocean Optics (Dunedin, FL, USA) USB2000 spectrophotometer attached to a laptop computer running OOIBASE32 (Ocean Optics). Reflectance spectra of different areas of the anemone illuminated with an external UV lamp were measured underwater at a 45° angle using a 200 µm bifurcated UV⁄visible optic fibre connected to Ocean Optics (Dunedin, FL, USA) USB2000 spectrophotometer (housed in a custom-built waterproof container), and analyzed later with the software Spectrasuite (Ocean Optics). For all measurements, a Spectralon 99% white reflectance standard was used to calibrate the percentage of light reflected at each wavelength from 300 to 800 nm. At least ten measurements per area and individual were taken and subsequently averaged. As no difference in spectral reflectance was visible between specimen of *A. akindynos*, measurements were averaged.

Visual modeling

*(a) Quantum catch equations*

Quantum catch of horizontal radiance is assumed to be independent of viewing distance and for a given photoreceptor i can be calculated from

$$Q_{rad,i} = k_i \int_{300nm}^{750nm} I_{rad,\lambda}\, L_\lambda A_{\lambda,i} d\lambda \qquad (6)$$

where $I_{rad,\lambda}$ is the sidewelling radiance, $L_\lambda$ is the lens transmission, $A_{\lambda,i}$ is the wavelength dependent photoreceptor absorptance, and $k_i$ is the von Kries correction for color constancy:

$$k_i = \frac{1}{\int_{300nm}^{750nm} I_{irrad\ ,\lambda} L_\lambda A_{\lambda.i} d\lambda} \qquad (7)$$

Here, we include this normalization for consistency with previous studies and for plotting the quantum catches in a trichromatic visual space. However, this correction does not impact the final discrimination calculations for either color or luminance, as comparisons between targets involve ratios of quantum catch, where the von Kries factor $k_i$ cancels out.

The quantum catch of a receptor i that views a target with reflectance spectra $R_\lambda$ illuminated by the sidewelling (or downwelling) irradiance, at zero viewing distance is given by

$$k_i = \frac{1}{\int_{300nm}^{750nm} I_{irrad\ ,\lambda} L_\lambda A_{\lambda.i} d\lambda} \qquad (8)$$

For simplicity, we do not include the effects of viewing distance which will act to decrease the contrast values due to scattering.

*(b) Visual discrimination equations*

Using the quantum catch at the target, we can calculate the luminance contrast:

$$LumContrast_{Noise} = \frac{\ln\left(\frac{Q_1}{Q_2}\right)}{\omega} \qquad (9)$$

where $Q_1$ and $Q_2$ are either two targets or one target compared to the horizontal radiance. These contrasts can be calculated for a single receptor, as luminance is often dominated by one receptor type. We then vary the peak sensitivity of that receptor to determine the wavelength at which contrast is maximum. The signal is then compared to receptor noise, $\omega$ (see below).

To determine color discrimination, we first calculate the signal contrast between two objects for each photoreceptor i as the log of the quantum catch ratio when comparing targets 1 and 2:

$$\Delta f_i = ln \frac{Q_{i,target\ 1}}{Q_{i,target\ 2}} \qquad (10)$$

These are then combined to include the input of all three receptors as

$$\Delta S = \left( \frac{\omega_1^2\ (\Delta f_2 - \Delta f_3)^2 + \omega_2^2\ (\Delta f_3 - \Delta f_1)^2 + \omega_3^2\ (\Delta f_1 - \Delta f_2)^2}{(\omega_1 \omega_2)^2 + (\omega_1 \omega_3)^2 + (\omega_2 \omega_3)^2} \right)^{1/2} \qquad (11)$$

where the noise value, $\omega_i$ for receptor i depends on the Weber fraction for a single receptor, $\nu_l$ taken to be the L cone, and the relative number density of photoreceptor i as compared to the L cone [34]:

$$\omega_i = \nu_i \sqrt{\frac{n_L}{n_i}} \qquad (12)$$

Retinal wholemount preparation

*(a) For photoreceptors and ganglion cells topography*

After fixation of retinas, radial cuts were performed in order to flatten the eye and subsequently the retina *in toto* onto a glass slide. The orientation of the retina was kept by referring to the position of the falciform process that ends ventrally. The sclera and choroid were gently removed, and the retina bleached overnight in a solution of 3% hydrogen peroxide in 0.1 M PB. For photoreceptor analysis, retinas were wholemounted (photoreceptor layer up) in 100% glycerol on a microscopic slide. For ganglion cells analysis, the retinas were wholemounted, ganglion cell layer facing up, on a gelatinized slide and left to dry overnight in formalin vapor to improved fixation and cell differentiation [4–6]. Wholemounts were then stained in 0.1 % cresyl violet following the protocol of Coimbra et al. [5] and finally mounted with Entellan New (Merck). Possible shrinkage during staining was considered negligible and if present confined to the retina margin, since the retinal wholemount was attached to the slide during the entire staining process [5].

*(b)* For in situ *analyses on wholemount retinas*

After eyes were enucleated and the cornea and lens were removed, the vitreous was dissolved enzymatically, directly in the eye cup, by treatment with hyaluronidase (Sigma, 200 U/ml) and collagenase (Sigma, 350 U/ml) in PBS for up to 30 min at room temperature. The retinas were then rinsed, dissected out of the eye cups and the retinal pigment epithelium removed mechanically using a jet of PBS. The retina wholemounts were then pinned down in a petri dish and fixed overnight in a solution of 4% paraformaldehyde in 0.1 M PBS (100nM PB with 5% sucrose), rinsed in PBS, put shortly in 70% methanol, and stored in 100% methanol until further processing.

Fluorescent *in situ* hybridization (FISH)

*(a) FISH protocol*

Following previously described methods [7–9], probes were labelled with DIG or Fluorescein (Roche DIG/Fluorescein RNA Labeling Mix, Sigma Aldrich), tagged with Alexa Fluor 594 and 488 dyes respectively (Invitrogen), and the signal was enzymatically augmented with sequential tyramide signal amplification (TSA amplification kits, Invitrogen). Retinas were finally mounted, photoreceptor side down, on coverslips in 70% glycerol/PBS.

*(b) Image acquisition of labeled opsins*

Whole retina scans for each dual-labelled opsin pair was performed using a spinning-disk confocal microscope, consisting of a Nikon Ti-E (Nikon Instruments Inc.) equipped with a Diskovery spinning-disk platform (Spectral Applied Research) and Zyla 4.2 sCMOS cameras (Andor). NIS Elements (Nikon Instruments Inc.) was used to perform multi-channel 3D tiled imaging with a CFI Plan Apochromat VC 20x objective (N.A. 0.75, W.D. 1.00mm) using a step size of 1.2µm. A water immersion CFI Apo Lambda S 40X objective (N.A. 1.25, W.D. 0.18mm)

was used to obtain high resolution images of selected regions of the retina. All scans were exported as TIFs and further processed (merging of colour channels, adjusting of brightness) with ImageJ 1.8.0_66 (National Institutes of Health, USA).

Stereological analyses and topographic map construction

*(a) Photoreceptor and ganglion cell densities*

Cells were randomly and systematically counted with a counting frame of 50 x 50 µm and a grid ranging from 310 x 310 µm to 510 x 510 µm using the Stereo Investigator software (Microbrightfield, USA). For ganglion cells analysis, sub-sampling using the same counting frame but a smaller grid of half the size of the original grid was also performed in the area of highest density to verify the peak density estimate.Cells counted using a x63 oil objective (numerical aperture 1.40). The counting frame and grid size were carefully chosen to maintain the highest level of sampling and achieve an acceptable Schaeffer coefficient of error (CE). The CE is a measure of the accuracy of the total number of cell estimates and is considered acceptable below 0.1 [10,11]. The grid was adjusted for all individuals to take into consideration the variation in total length between specimens and allow sampling of around 200 sites per retinas.

Single cones and double cones were counted separately and simultaneously using two different markers to generate data for single cones alone, double cones alone, and the two cell types combined (total cones). Ganglion cells were arranged in a single layer within the ganglion cell layer. Two other cell types are also found within the ganglion cell layer, amacrine cells and glial cells and are usually easily distinguished from ganglion cells using cytological criteria alone [12,13]. However, in the case of *A. akindynos*, ganglion cells and amacrine cells were difficult to tell apart, especially in higher density areas. For this reason, amacrine cells were included in the ganglion cell counts and only glial cells were excluded. While the inclusion of amacrine cells in the analysis should not influence the overall topography [5,14–17], it will contribute to a slight overestimation of the peak density of ganglion cells and ultimately to a slight overestimation of spatial resolving power.

*(b) Opsin gene densities*

Widefield 3D image stacks of opsin genes labelled with Alexa Fluor 488 and Alexa Fluor 594 were acquired on a Zeiss AxioImager Z1 (Carl Zeiss), fitted with an ORCA-ER (Hamamatsu Photonics K.K.) and 0.63x C-Mount, using a EC Plan-Neofluar 40x objection (N.A. 0.75, W.D. 0.71 mm) with sufficient range to cover the maximum retinal thickness and step size of 2.5 µm. Image stacks were further processed with Image J 1.8.0_66 (National Institutes of Health, USA). To avoid counting regions damaged during the retina wholemount preparation protocol, a counting frame of 550 x 550 pixels (140.8 x 140.8 $\mu m^2$) was manually positioned within each image stack in a location containing no holes or damaged tissue. As the 200 sites per retina were collected using systematic random sampling, we do not consider this additional manual region cropping to negatively impact the reliability of the analysis.

For topographic map reconstructions we used R and a custom script adapted from Garza-Gisholt et al [18]. Briefly, the information about the contour of the retina and location of the 200 images taken for the analysis, were exported from Stereo Investigator software and mapped using R. Subsequently, opsin densities (in cells/$mm^2$) for each site was added manually on the map using Adobe Illustrator CC 2018. The entire file was then saved as a scalable vector graphics (.svg) file and a heatmap of expression was constructed in R using the Gaussian kernel smoother from the Spatstat package with a sigma value adjusted to 30.

Spatial revolving power estimation

The upper limit of the spatial resolving power (SRP) in cycles degrees i.e., visual acuity, was estimated using the ganglion cell peak density as described by Collin & Pettigrew [19]. Briefly, assuming that the focal length *f* for teleost fish is 2.55 times the radius of the lens (Matthiessen ratio [20]), the angle subtending 1 mm on the retina (angle $\alpha$) can be calculated as follow:

$$\alpha = \arctan(1/f) \qquad (2)$$

Knowing $\alpha$, the peak density of ganglion cells (PDG in cells/mm) and the fact that two ganglion cells are needed to distinguish a visual element from its neighbor, the SRP in cycles per degree can be calculated as follow:

$$SRP = (PDG/\alpha)/2 \qquad (3)$$

The maximum distance (Dist) at which an individual could resolve a specific object of a given size (S), was calculated as described by Coimbra et al. [21].

$$Dist = S/\tan \qquad (4)$$

Where $\beta$ is the minimum separable angle:

$$\beta = 1/SRP \qquad (5)$$

## Tables and Figures

**Table S1.** Genbank accession numbers of *Amphiprion akindynos* opsin sequences and transcriptomes sequenced in this study (BioProject ID: PRJNA547682). Labeling of specimen "#" refer to table S1.

| opsin gene | Accession number | ID specimen | Accession number |
|---|---|---|---|
| SWS1 | MN098320 | female #4 | SAMN11982617 |
| SWS2B | MN098319 | female #8 | SAMN11982614 |
| RH2B | MN098321 | female #10 | SAMN11982621 |
| RH2A | MN098322 | female #11 | SAMN11982622 |
| LWS | MN098324 | male #5 | SAMN11982618 |
| RH1 | MN098323 | male #6 | SAMN11982616 |
| | | immature #5 | SAMN11982623 |
| | | immature #6 | SAMN11982619 |
| | | immature #7 | SAMN11982615 |
| | | immature #8 | SAMN11982620 |

**Table S2** Summary of beta regression showing that opsin gene expression does not correlate to sex or size in *Amphiprion akindynos* (n=10). Applying for Bonferroni correction for multiple comparison (n=2), p-Values ≤ 0.025 were considered significant.

| | ***SWS1*** | ***SWS2B*** | ***RH2B*** | ***RH2A*** | ***LWS*** |
|---|---|---|---|---|---|
| *Sex* | 0.273 | 0.209 | 0.47869 | 0.641 | 0.423 |
| *Size* | 0.89472 | 0.89472 | 0.311 | 0.216 | 0.0991 |

**Table S3** Overview of visual pigment $\lambda_{max}$-measurements gained by microspectrophotometry (MSP), and best matching $\lambda_{max}$-estimates of opsin genes, as well as $\lambda_{max}$-data used for visual models of *Amphiprion akindynos*.

| **Visual pigment $\lambda_{max}$ [nm] gained from MSP** | **Rod** | **Single cone** | | | | |
|---|---|---|---|---|---|---|
| | | **UV-violet** | | **blue** | **green** | **red** |
| Mean MSP (3 specimens) | 498 ± 4 (n=23) | 400 ± 3 (n=6) | | 498 ± 4 (n=12) | 520 ± 5 (n=13) | 541 (n=1) |
| | | Coexpression SWS1/SWS2B | | | | |
| **Opsin $\lambda_{max}$ [nm] estimate** | **RH1** | **SWS1** | **SWS2B** | **RH2B** | **RH2A** | **LWS** |
| *Based on cichlids* | 496 | | 408 | | 518 | 554 |
| *Based on other damselfish* | - | 370 | - | 498 | 516/523 | |
| **$\lambda_{max}$ [nm] used for visual models** | - | 370 - 408 | | 498 | 520 | - |

**Table S4** Overview of sites and site effects considered for $\lambda_{max}$-calculations of *Amphiprion akindynos*. Site numbers refer to the corresponding bovine RH1 (GenBank Accession No.: NP_001014890.1) as well as to *A. akindynos* opsins. Calculations are based on opsin sequence comparison to reference species with known pure protein spectral absorbance gained from *in-vitro* opsin protein expression studies (*Oreochromis niloticus* [29], *Maylandia zebra* [28], and *Oryzias latipes* [35]), and/or to other damselfish species having a very similar sequence in known tuning sites and with known spectral absorbances gained from MSP (*Pomacentrus amboinensis* [31], *P. coelestis*, and *Dascyllus trimaculatus* [30]). Only AA changes at retinal chromophore binding pocket sites, as well as previously determined tuning sites [27,36–41] are shown.

| **RH1** | variable AA sites in binding pocket or at known tuning sites | | |
|---|---|---|---|
| *O. latipes* RH1 502nm | D | S | S |
| *O. niloticus* RH1 | D | S | S |
| *M. zebra* RH1 | D | A | A |
| *A. akindynos* RH1 | N | S | S |
| *A. akindynos* RH1 site # | 83 | 298 | 299 |
| *Bovine* RH1 site # | 83 | 298 | 299 |
| Known tuning sites in same or other opsins | D83N (RH1)=-6<br>N83D (RH1)=+2 | S298A (SWS1)=0 | S299A (RH1)=-2 |

| | Estimated tuning effect [nm] based on *O. latipes* 502nm | | | Estimate [nm] |
|---|---|---|---|---|
| *A. akindynos* RH1 | -6 | 0 | 0 | 496 |

RH1 in *A. akindynos* was estimated to be maximally sensitive at 496 nm, based on *O. latipes* having a $\lambda_{max}$ of 502 nm [35]. Only one RH1 amino acid differed between species at a known tuning site. This site D83N is reported to shift $\lambda_{max}$ to shorter wavelengths (-6 nm,[27]).

| **SWS1** | variable AA sites in binding pocket or at known tuning sites | | | | | | |
|---|---|---|---|---|---|---|---|
| *O. latipes* SWS1 356nm | F | F | L | A | A | S | |
| *O. niloticus* SWS1 360nm | L | F | L | A | A | S | |
| *M. zebra* SWS1 368nm | F | F | L | S | S | A | |
| *P. amboinensis* 370nm | F | C | F | S | S | S | |
| *A. akindynos* | F | C | F | S | S | S | |
| *A. akindynos* SWS1 site # | 41 | 42 | 89 | 107 | 111 | 291 | |
| *Bovine* RH1 site # | 48 | 49 | 96 | 114 | 118 | 298 | |
| Known tuning sites in same or other opsins | | F49V (SWS1)<br>F49L (SWS1)<br>S49F (RH2)<br>S49A (RH2) | Y96V (RH1) | A114G (SWS1)=+x | A118T (SWS1)=+3<br>T118G (SWS2)=15<br>T118A (RH1)=-16 | S298A (SWS1)=0 | |
| | Estimated tuning effect [nm] based on *M. zebra* 368nm | | | | | | Estimate [nm] |
| *A. akindynos* SWS1 | 0 | ? | ? | 0 | 0 | 0 | ? |
| | Estimated tuning effect [nm] based on *P. amboinensis* 370nm | | | | | | Estimate [nm] |
| *A. akindynos* SWS1 | 0 | 0 | 0 | 0 | 0 | 0 | 370 |

SWS1 was estimated to be sensitive at 370 nm based on *P. amboinensis*, having the same sequence structure as *A. akindynos*. Amino acid structure of *M. zebra* was closest to the one in *A. akindynos*, and differed at three sites. Of those, A298S was expected to have no tuning effect as S298A has been shown not to effect $\lambda_{max}$ [40], while the tuning effect of F49C and L96F together could add up to the +2 nm shift when compared to *P. amboinensis*.

| **SWS2B** | variable AA sites in binding pocket or at known tuning sites | | | | | | |
|---|---|---|---|---|---|---|---|
| *O. latipes* SWS2B 405nm | L | V | A | C | S | F | Y |
| *O. niloticus* SWS2B 423nm | F | F | T | C | A | Y | W |
| *M. zebra* SWS2B 423nm | F | F | T | C | A | Y | W |
| *A. akindynos* SWS2B | F | F | S | F | A | Y | Y |
| *A. akindynos* SWS2B site # | 49 | 52 | 124 | 169 | 174 | 209 | 271 |
| *Bovine* RH1 site # | 43 | 46 | 118 | 163 | 168 | 203 | 265 |
| Known tuning sites in same or other opsins | | F46V/L (SWS2)<br>F46T (SWS1) | T118G (SWS2)=15<br>T118A (RH1)=-16 | | | | W265Y (RH1)=-15<br>Y265W (SWS1)=+10 |

| | | | | | | | | |
|---|---|---|---|---|---|---|---|---|
| | | | A118T (SWS1)=+3 | | | | F261Y (LWS)=+6 | |
| | Estimated tuning effect [nm] based on *M. zebra* and *O. niloticus* 423nm | | | | | | | Estimate [nm] |
| *A. akindynos* SWS2B | 0 | 0 | 0 | 0 | 0 | 0 | -15 | 408 |

SWS2B in *A. akindynos* was estimated, based on *O. niloticus* and *M. zebra* SWS2B of 423 nm [28,29], to be maximally sensitive at 408 nm. Three amino-acid residues in retinal binding pocket or known tuning sites were different between the anemonefish and cichlid sequences. Of these, only W265Y is a known tuning site, shifting $\lambda_{max}$ to shorter wavelengths (-15 nm, [42]). Site 118 is a known tuning site [27] but as T118S has no polarity change, we therefore assumed this change not to affect $\lambda_{max}$ tuning in *A. akindynos*. Finally, C163F is not a known tuning site, and is therefore also assumed not to affect $\lambda_{max}$ tuning.

| **RH2B** | variable AA sites in binding pocket or at known tuning sites | | | | | | | |
|---|---|---|---|---|---|---|---|---|
| *O. niloticus* RH2B 472nm | A | M | F | V | I | S | M | |
| *M. zebra* RH2B 484nm | A | I | S | V | I | S | M | |
| *P. coelestis* 490nm | G | M | F | I | C | G | L | |
| *D. trimaculatus* 490nm | A | M | L | I | C | G | L | |
| *A. akindynos* RH2B | G | M | V | I | C | G | L | |
| *A. akindynos* RHB site # | 42 | 45 | 47 | 49 | 50 | 110 | 208 | |
| *Bovine* RH1 site # | 41 | 44 | 46 | 48 | 49 | 109 | 207 | |
| Known tuning sites in same or other opsins | | M44T (SWS2) | F46V (SWS2)=+8<br>F46T (SWS1)<br>F46L(SWS2) | | S49F (RH2)<br>S49A (RH2)<br>F49V (SWS1)<br>F49L (SWS1) | V109A (SWS1)<br>A109G (SWS2) | M207L (RH2)=-6<br>L207M (RH2)=+6<br>L207I (SWS2)=-6 | |
| | Estimated tuning effect [nm] based on *O. niloticus* 472nm | | | | | | | Estimate [nm] |
| *A. akindynos* RH2B | 0 | 0 | +8 | 0 | +8 | | -6 | 488 |
| | Estimated tuning effect [nm] based on *P. coelestis* 490nm | | | | | | | Estimate [nm] |
| *A. akindynos* RH2B | 0 | 0 | +8 | 0 | 0 | 0 | 0 | 498 |
| | Estimated tuning effect [nm] based on *D. trimaculatus* 490nm | | | | | | | Estimate [nm] |
| *A. akindynos* RH2B | 0 | 0 | 0 | 0 | 0 | 0 | 0 | 490 |

RH2B in *A. akindynos* was estimated to be maximally sensitive at 488 nm based on *O. niloticus*, respectively 494/8 nm based on *P. coelestis* and *D. trimaculatus*. Comparing RH2B sequences between *A. akindynos* and *O. niloticus* (472 nm [28]), six amino acid sites differed, adding up to a maximal absorbance of 488 nm. The two sites A41G and V48I were considered to not shift $\lambda_{max}$ as both changes were between amino acids with non-polar side chains. Site 46 is a known tuning site for a positive tuning shift for F46T/L in SWS proteins [27]; F46V, as seen in *A. akindynos*, is also present in SWS2B of *O.latipes* (405 nm) when compared to SWS2B of *Lucania goodei* (397 nm), possibly causing the +8 nm shift [41]. We also suggest the F46V change in RH2B to cause a_+8 nm tuning effect. I49C and S109G are no known tuning sites, however, they were previously [43] thought to be the underlying substitutions adding up to the 8 nm sensitivity difference observed between *O. niloticus* (472 nm [28]) and *P. amboinensis* (480 nm [31]) RH2B. As the same substitutions were seen in *A. akindynos*, we also assumed them to account for a +8 nm shift. The last site difference is site M207L, a tuning site known for a -6 nm shift [39]. When estimates were based on the RH2B sequence of. *D. trimaculatuss* (490 nm), no sites differed, resulting in a $\lambda_{max}$ of 490 nm. When estimates were based on the RH2B sequence of. *P.coelestis* (480 nm), the only variable amino acid site is M207L (+6 nm), resulting in a $\lambda_{max}$ of 498 nm.

| **RH2A** | variable AA sites in binding pocket or at known tuning sites | |
|---|---|---|
| *O. niloticus* RH2Aα 528nm | F | |
| *O. niloticus* RH2Aβ 518nm | L | |
| *M. zebra* RH2Aα 528nm | F | |
| *M. zebra* RH2Aβ 519nm | L | |
| *P. amboinensis* 523nm | I | |
| *D. trimaculatus* 516nm | I | |
| *A. akindynos* RH2A | I | |
| *A. akindynos* RH2A site # | 166 | |
| *Bovine* RH1 site # | 158 | |
| Known tuning sites in same or other opsins | F158L/I(RH2A)=-~10 | |
| | Estimated tuning effect [nm] based on *M. zebra* and *O. niloticus* RH2Aalpha 528nm | Estimate [nm] |
| *A. akindynos* RH2A | -10 | 518 |
| | Estimated tuning effect [nm] based on *P. amboinensis* 523nm and *D. trimaculatus* 516nm | Estimate [nm] |
| *A. akindynos* RH2A | 0 | 516/523 |

RH2A was estimated to be maximally sensitive at 518 nm, based on RH2Aα of *M. zebra* and *O. niloticus* having a $\lambda_{max}$ of 528nm [28,29]. A blue-shift of around 10 nm in $\lambda_{max}$ of RH2Aβ compared to RH2Aα in both cichlid species is likely caused by a F158L substitution, A similar substitution from an aromatic group to a non-polar residue (F158I) in *A. akindynos* was estimated to have a similar tuning effect. This estimate of 518 nm falls within the range of a $\lambda_{max}$ estimate of 516 and 523 nm based *D. trimaculatus* (516 nm) and *P. amboinensis* (523 nm), both having I158 as seen in A. akindynos.

| **LWS** | variable AA sites in binding pocket or at known tuning sites | | | | |
|---|---|---|---|---|---|
| *O. latipes* LWSA/B 561/62nm | S | H | Y | T | A |
| *O. niloticus* LWS 561nm | S | A | Y | T | A |
| *M. zebra* LWS 554nm | A | H | Y | T | A |
| *A. akindynos* LWS | A | H | Y | T | A |
| *A. akindynos* LWS site # | 177 | 194 | 274 | 282 | 305 |
| *Bovine* RH1 site # | 164 | 181 | 261 | 269 | 292 |

| Known tuning sites in same or other opsins | S164A (LWS)=-7<br>A164S (LWS)=+6 | H181Y (LWS) | Y261F (LWS)=-10<br>F261Y (LWS)=+6<br>Y261F (RH1)=-8<br>F261Y (RH1)=+10<br>Y261F (SWS2)=-5 | A269T (LWS)=+10<br>T261A (LWS)=-16<br>A269T (RH1)=+14<br>A269T (SWS2)=+6 | S269A=+28<br>A269S (SWS2)=-8<br>S269A (SWS1)=0<br>A269S (RH1)=-10<br>S269A (RH1)=8 | |
|---|---|---|---|---|---|---|
| | Estimated tuning effect [nm] based on *M. zebra* 554nm | | | | | Estimate [nm] |
| *A. akindynos* LWS | 0 | 0 | 0 | 0 | 0 | 554 |
| | Estimated tuning effect [nm] based on *O. niloticus* 561nm | | | | | |
| *A. akindynos* LWS | -7 | 0 | 0 | 0 | 0 | 554 |

LWS in *A. akindynos* was estimated - based on *O. niloticus* and *M. zebra* LWS of 561 nm and 554 nm, respectively [28,29] – to be maximally sensitive at 554 nm. *A. akindynos* and *M. zebra* LWS protein sequence did not differ in retinal binding pocket or known tuning sites. In comparison with *O. niloticus* LWS, two sites differed, but only S164A is known to shift spectral sensitivity in LWS opsin to shorter wavelengths (-7 nm, [27]).

**Table S5** Summary of *Amphiprion akindynos* specimens from this study, their source, size, sex, if applicable origin from anemone, type of analysis they were used for, and if applicable which eye was used. SC = single cone, DC = double cone, PR = photoreceptor, GC = ganglion cells, In situ = fluorescent *in situ* hybridization, RNAseq = RNA sequencing, MSP = microspectrophotomerty, lens = lens transmission, spec = spectral reflectance. * Most likely sex/life stage.

| Sex | Individual | Size (TL in cm) | Anemone | Source | Eye used | Analysis type |
|---|---|---|---|---|---|---|
| Female | 1 | 10.9 | A | Lizard Island | Left | In situ SC |
| | 2 | 9.1 | B | Lizard Island | Right<br>Left | In situ DC<br>LWS mapping |
| | 3 | n/a | n/a | Cairns Marine | Both | MSP |
| | 4 | 8.9 | C | Lizard Island | Left | PR mapping<br>Lens<br>RNAseq |
| | 5 | 8.8 | n/a | Cairns Marine | Left | GC mapping |
| | 6 | 8.8 | D | Lizard Island | | Spec |
| | 7 | 8.6 | E | Lizard Island | Left | LWS mapping |
| | 8 | 7.9 | F | Lizard Island | Right | RNAseq |
| | 9 | 7.3 | G | Lizard Island | Right | PR mapping<br>Lens |
| | 10 | 6.2 | H | Lizard Island | Right | RNAseq<br>Lens |
| | 11 | 4.6 | I | Lizard Island | Right | PR mapping<br>RNAseq |
| Male | 1 | 10.4 | E | Lizard Island | Left | LWS mapping |
| | 2 | 8.8 | n/a | Lizard Island | Both | MSP |
| | 3 | n/a | n/a | Cairns Marine | Both | MSP |
| | 4 | 8.4 | J | Lizard Island | Left | In situ SC |
| | 5 | 7.8 | C | Lizard Island | Left | PR mapping<br>Lens<br>RNAseq |
| | 6 | 7.8 | F | Lizard Island | Right | RNAseq |
| | 7 | 7.4 | | Lizard Island | Left | Lens |
| | 8 | 6.8 | D | Lizard Island | | Spec |
| | 9 | 6.5 | n/a | Cairns Marine | Left<br>Right | PR mapping<br>GC mapping |
| Immature | 1 | 6.8 | J | Lizard Island | Left | In situ SC |
| | 2 | 6.6 | E | Lizard Island | Left | In situ DC |
| | 3 | 5.9 | D | Lizard Island | | Spec |
| | 4 | 4.1 | n/a | Lizard Island | Left | PR and GC mapping<br>Lens |
| | 5 | 3.5 | I | Lizard Island | Right | RNaseq |
| | 6 | 3.2 | H | Lizard Island | Left | PR mapping<br>Lens<br>RNAseq |
| | 7 | 3.1 | F | Lizard Island | Right | RNAseq |
| | 8 | 2.4 | H | Lizard Island | Left | RNAseq<br>Lens |

**Table S6.** Primers used for probe template (length of at least 600 bases) design. RNA Polymerase promoter sequences (T7 resp. T3) were incorporated in primer sequences.

| opsin | primer | Sequence |
|---|---|---|
| SWS1 | SWS1_forward | 5-TAATACGACTCACTATAGGGCCACCTGTACGAGAACATCTCC-3’ |
| | SWS1_reverse | 5- AATTAACCCTCACTAAAGGGCCATTTCCATGATGCAGGCG -3’ |
| SWS2B | SWS2B_forward | 5’- TAATACGACTCACTATAGGGCTTCTGGATCCCCATCGCTC -3’ |
| | SWS2B_reverse | 5’- AATTAACCCTCACTAAAGGGGTGGCCAGTCGTAGGTCAAA -3’ |
| RH2B | RH2B_forward | 5’- TAATACGACTCACTATAGGGGCATGGTGGGCTATTTCTCCT -3’ |
| | RH2B_reverse | 5’- AATTAACCCTCACTAAAGGGCTCACCCACTCATCCATCCAA -3’ |
| RH2A | RH2A_forward | 5’- TAATACGACTCACTATAGGGCTGTCAACGGCTACTTCATTCTT -3’ |
| | RH2A_reverse | 5’- AATTAACCCTCACTAAAGGGGACACACATGCTCCCCCATA -3’ |
| LWS | LWS_forward | 5’- TAATACGACTCACTATAGGGAGGCCCCAACTACCACATTG -3’ |
| | LWS_reverse | 5’- AATTAACCCTCACTAAAGGGTGTAGATGGTGGCGCTCTTG -3’ |

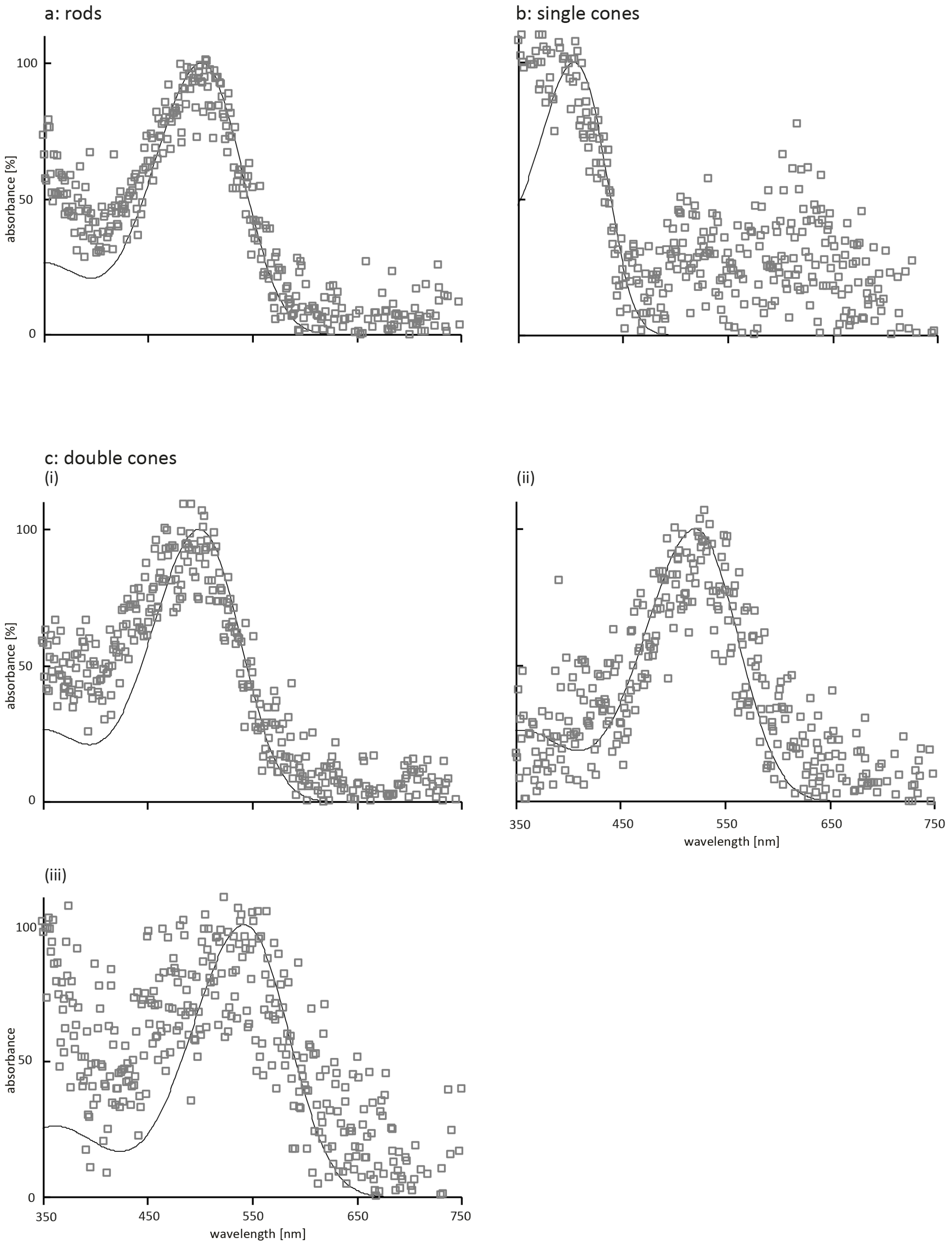


**Fig. S1:** Absorbance curves of rod and cone cells gained from microspectrophotometry (MSP). (a) $\lambda_{max}$ for rods was 498 ± 4 nm (n=23). (b) Single cone was 400 ± 3 nm (n=6). (c) Double cones were grouped according to their $\lambda_{max}$in was 498 ± 4 nm (n=12) (ii), 520 ± 5 nm (n=13), and 541 nm (n=1) (iii).

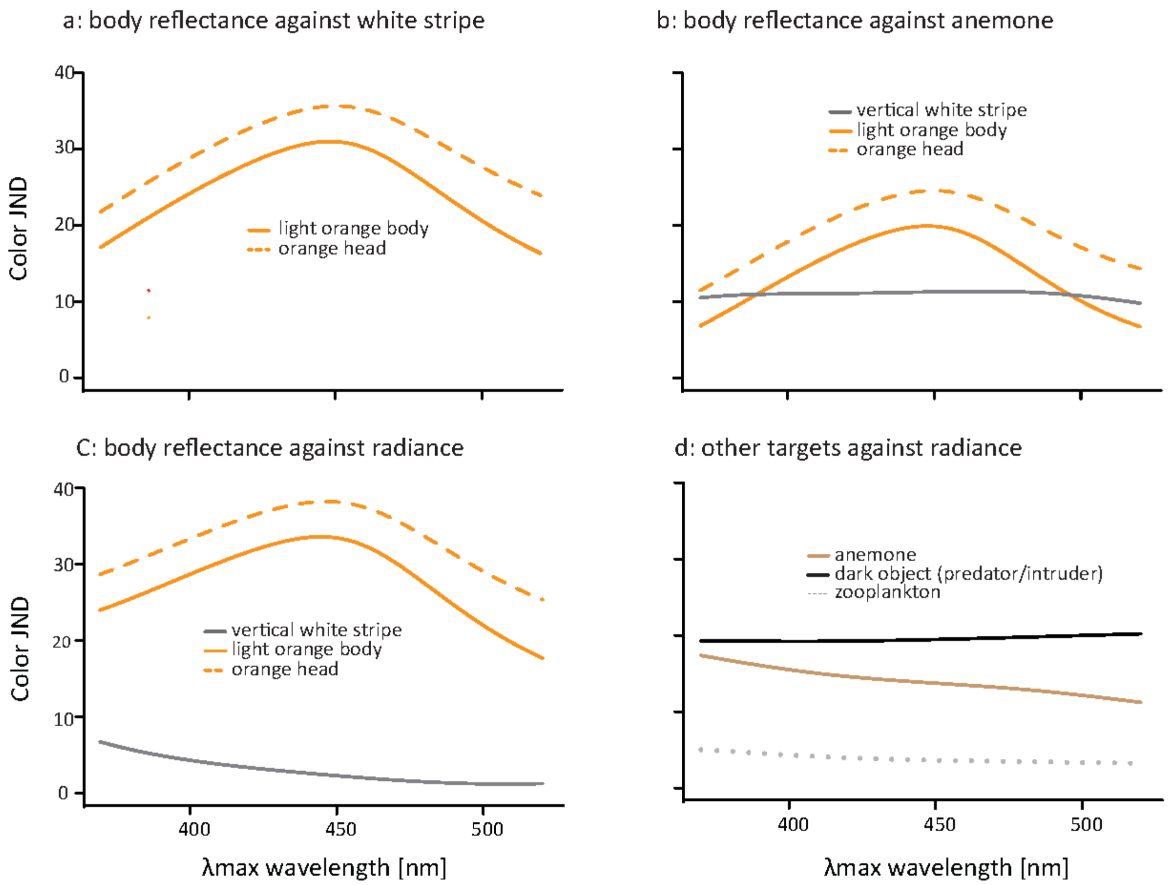


**Fig. S2:** Luminance contrast calculated for distinguishing pairs of targets as single visual pigment peak sensitivity ($\lambda_{max}$) is varied: (a) head orange (450 nm) or body orange (447 nm) versus white stripe. (b) Fish colors versus horizontal radiance: head orange (446 nm), body orange (444 nm) and white stripe (370 nm). (c) Fish colors versus anemone: head orange (449 nm), body orange (447 nm) and white stripe (464 nm). d: other targets compared to the horizontal radiance: anemone (370 nm), dark predator (520 nm) and zooplankton (370 nm).

a: Whole retinal scan illustrating double cone opsins
*RH2A* (green) and *RH2B* (magenta)

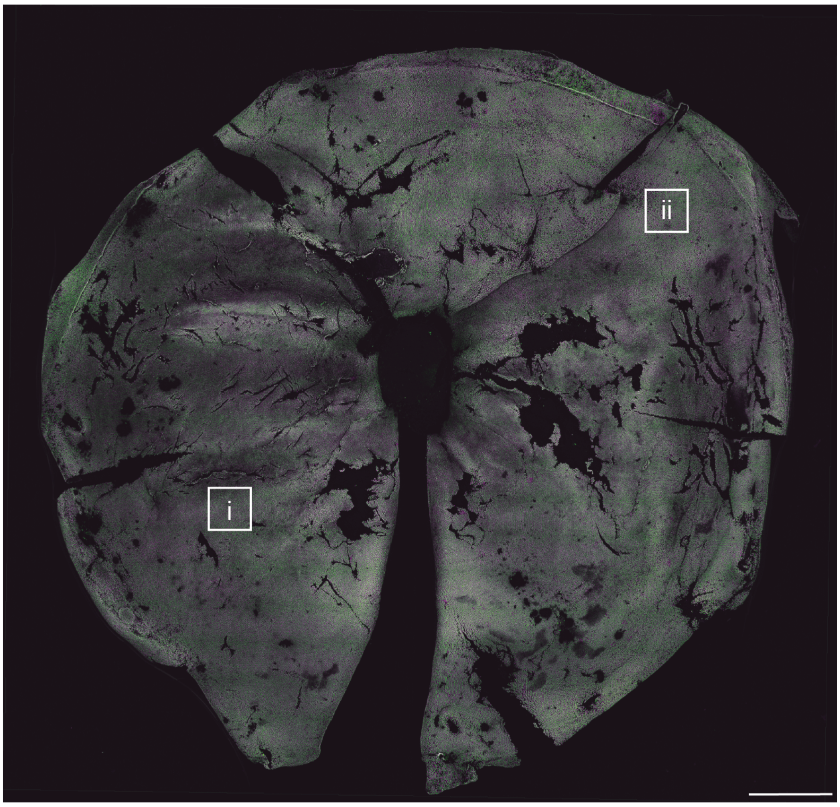


b: Whole retinal scan illustrating single cone opsins single cone opsins:
*SWS1* (green) and *SWS2B* (magenta)

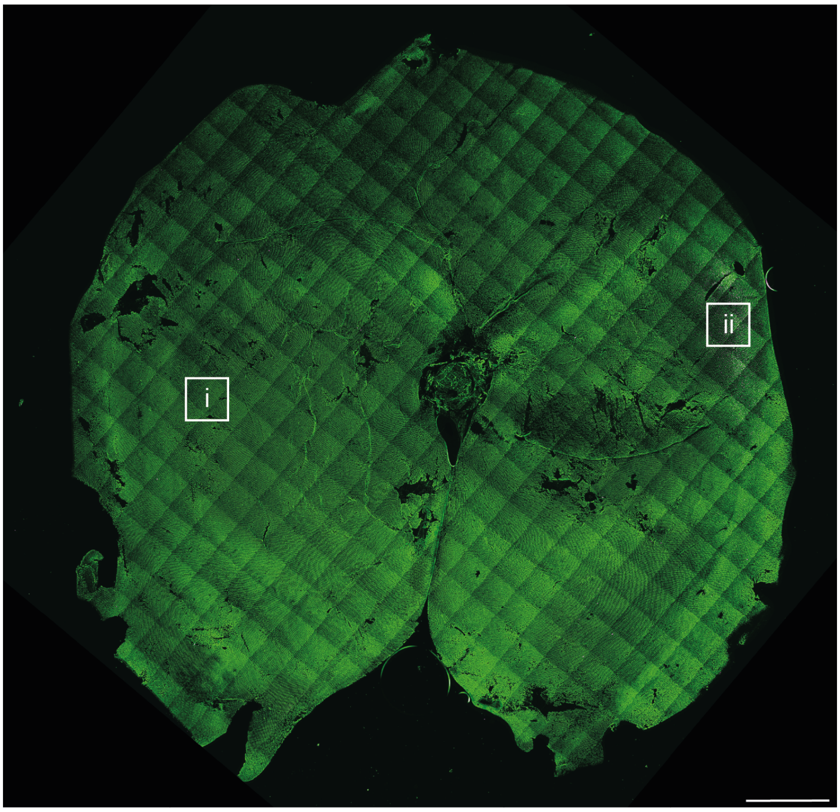


**Fig. S3:** Opsin expression in double (a) and single (b) cones revealed by fluorescent *in situ* hybridization (FISH) in wholemount retinas of *Amphiprion akindynos*. Whole retina scans reveal the expression patterns of *RH2A* (green) and *RH2B* (magenta) in double cones (a), and *SWS1* (green) and *SWS2B* (magenta) in single cones (b). Inserts i and ii refer to high resolution images shown in Figure 2b and c, respectively. Scale bar 100 µm.

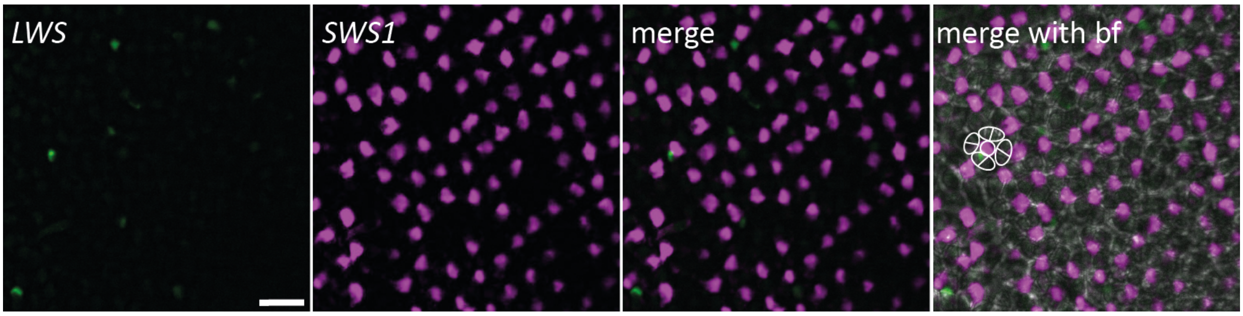


**Fig. S4:** Opsin expression of LWS and SWS1 revealed by fluorescent *in situ* hybridization (FISH) in wholemount retinas of *Amphiprion akindynos*. High resolution images show that LWS (green) is expressed in double cones, and SWS1 (magenta) in single cones. Scale bar 10 µm.

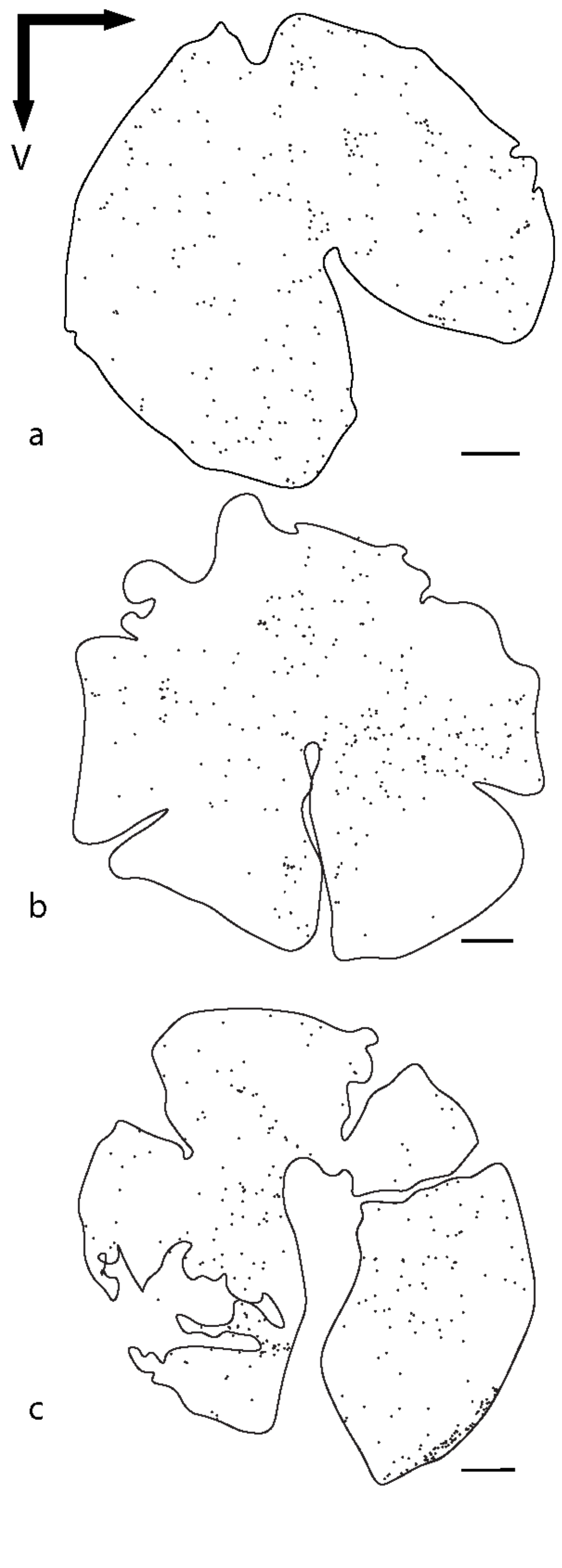


**Fig. S5:** Distribution of the photoreceptors expressing the *LWS* opsin gene, as revealed by FISH, in three individuals of *A. akindynos* of different sex/life stage: female (a), male (b) and immature (c). Each dot represent one labelled photoreceptor. The black arrow indicates the orientation of the retinas. T = temporal, V = ventral Scale bar 100 µm.

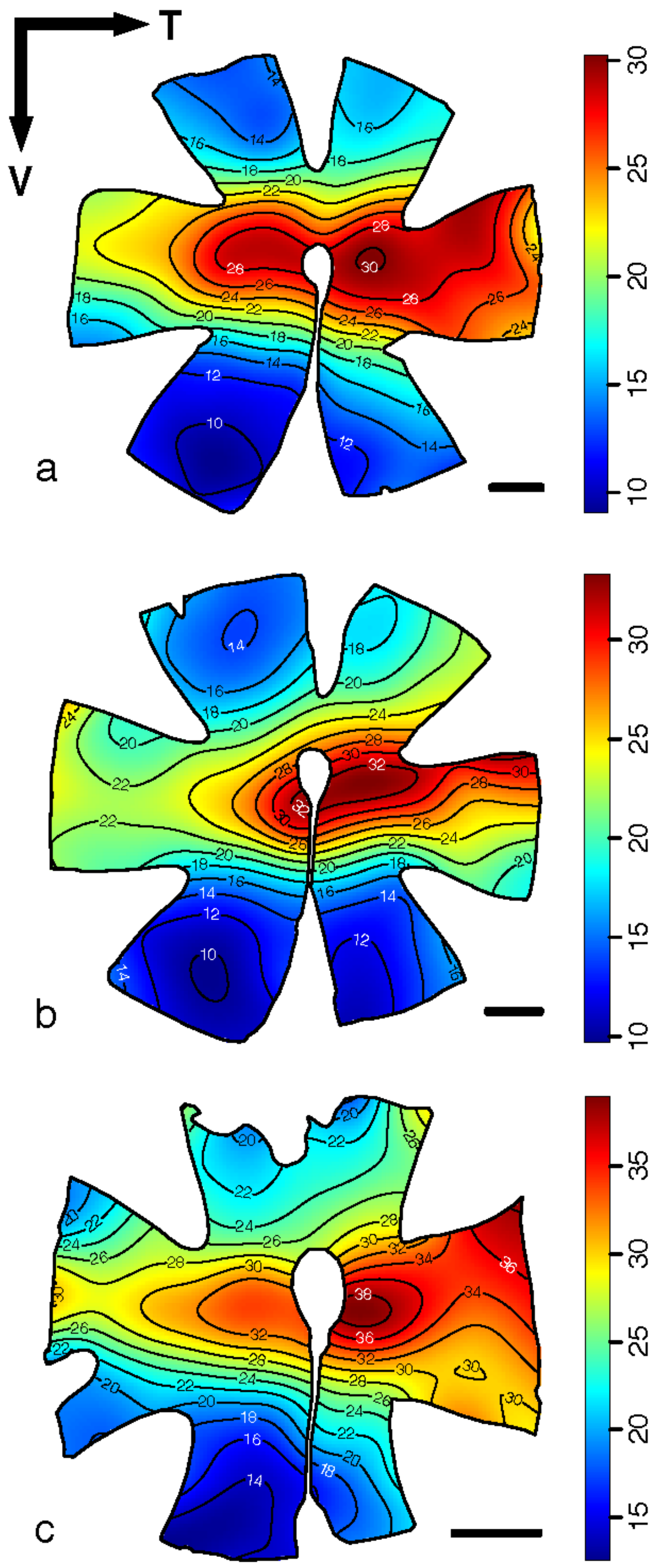


**Fig. S6:** Topographic distribution of ganglion cell densities in the retina of three individuals of *A. akindynos* of different sex/life stage: female (a), male (b) and immature (c). The black lines represent iso-density contours and values are expressed in densities x $10^3$ cells/$mm^3$. The black arrow indicates the orientation of the retinas. T = temporal, V = ventral. Scale bars: 1 mm.

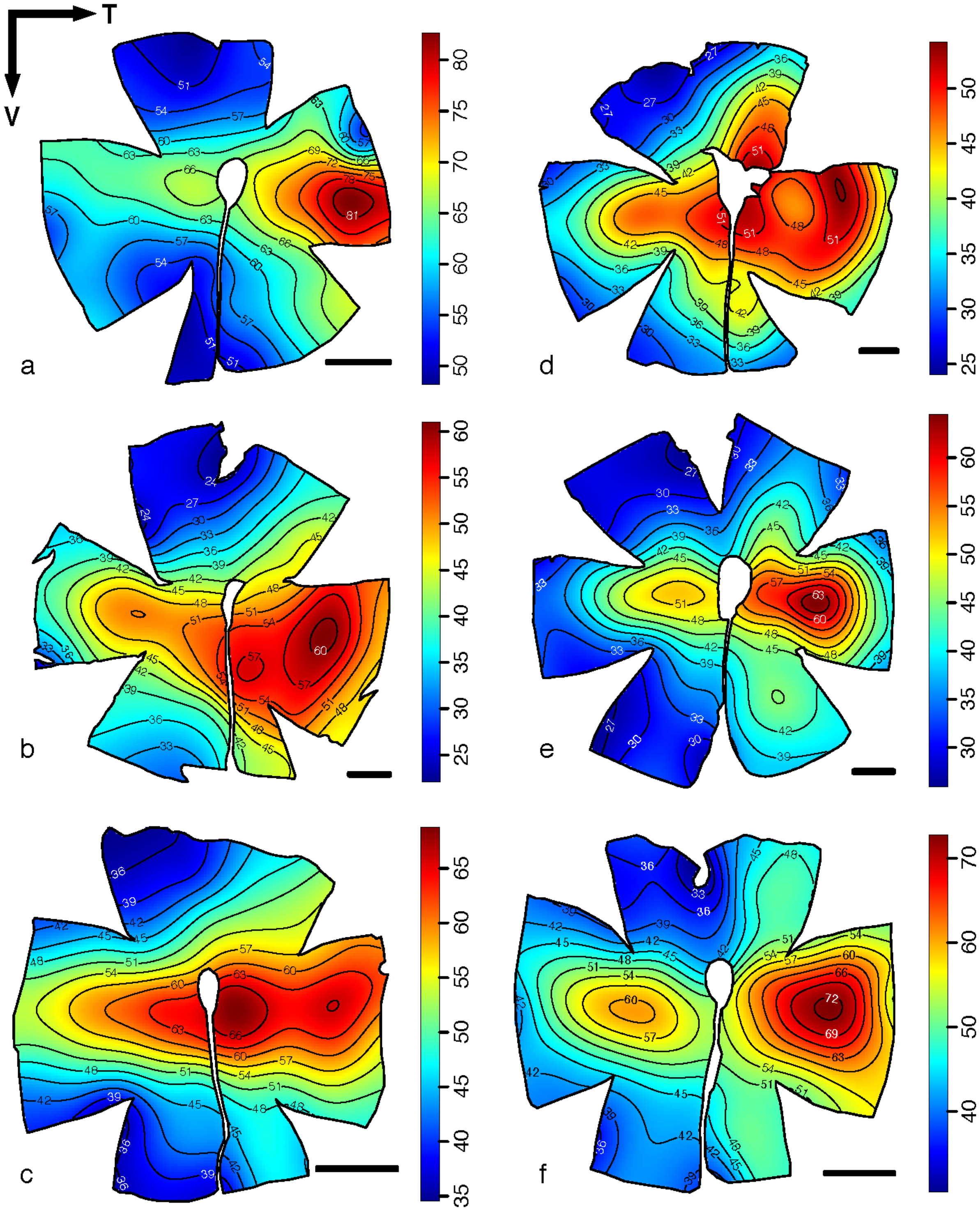


**Fig. S7:** Topographic distribution of total cone densities in the retina of six individuals of *A. akindynos* of different sex/life stage: female (a-b), male (c-d) and immature (e-f). The black lines represent iso-density contours and values are expressed in densities x $10^3$ cells/$mm^3$. The black arrow indicates the orientation of the retinas. T = temporal, V = ventral. Scale bars: 1 mm.

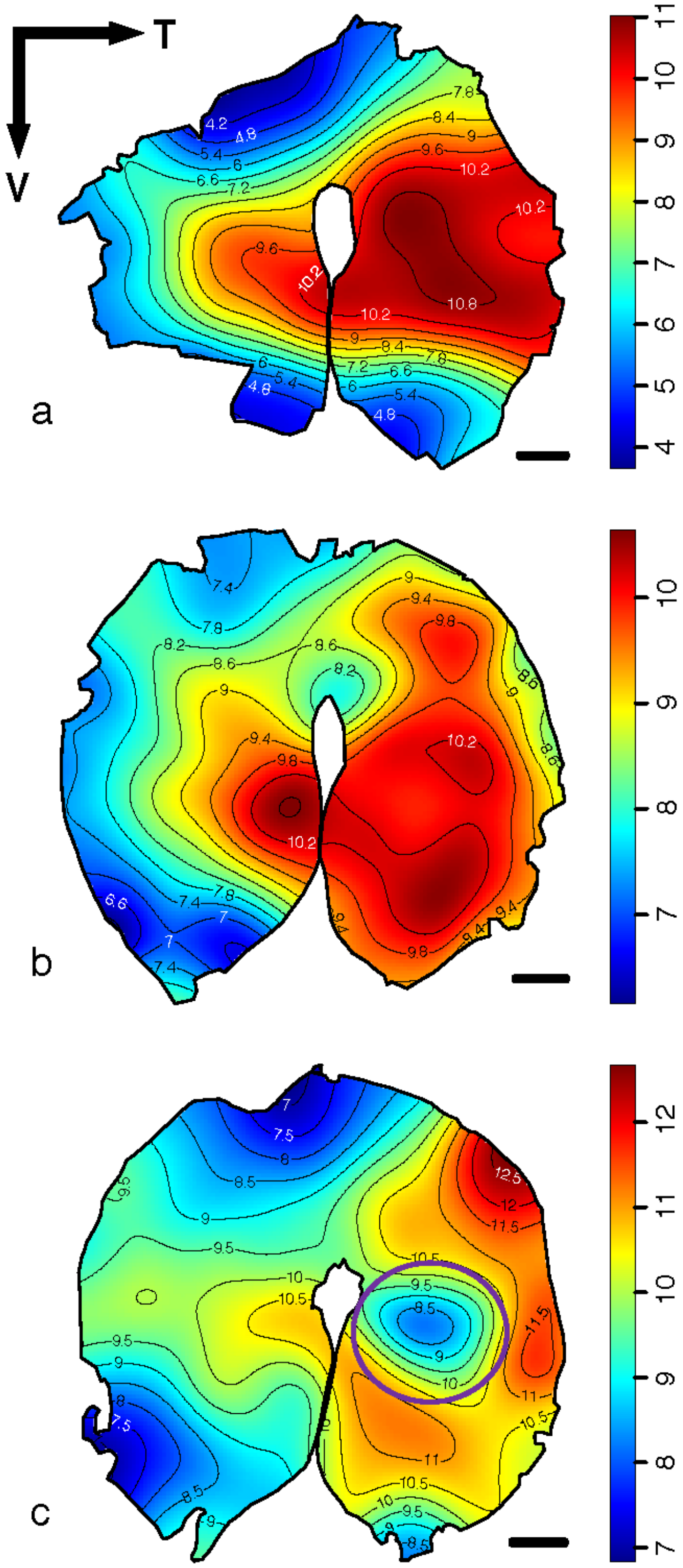


**Fig. S8:** Topographic distribution of SWS1 opsin densities in the retina of three individuals of *A. akindynos* of different sex/life stage: female (a), male (b) and immature (c). The black lines represent iso-density contours and values are expressed in densities x $10^3$ cells/mm$^3$. The black arrow indicates the orientation of the retinas. T = temporal, V = ventral. The purple circle in (c) highlights an under-labeled area, most likely due to a loss of photoreceptor cells during the FISH protocol. Scale bars: 1 mm.

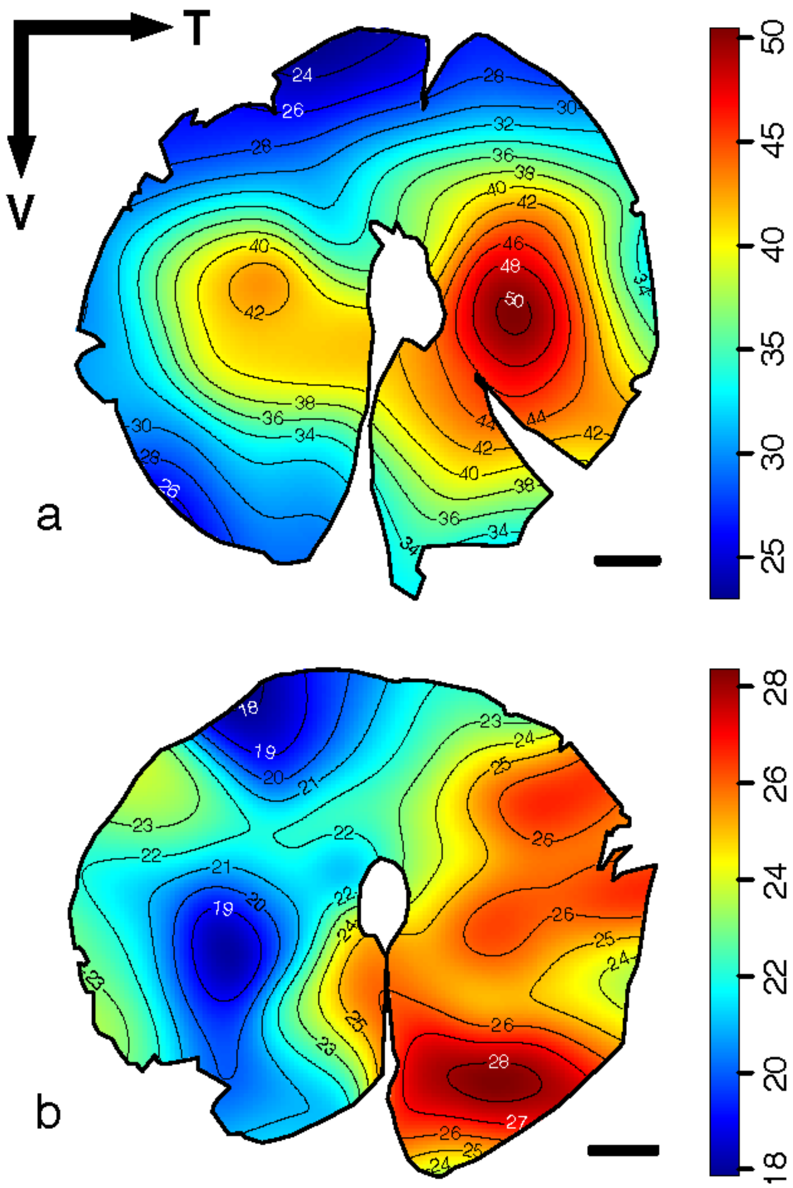


**Fig. S9:** Topographic distribution of RH2 opsin densities in the retina of two individuals of *A. akindynos* of different sex/life stage: female (a) and immature (b). The black lines represent iso-density contours and values are expressed in densities x $10^3$ cells/$mm^3$. The black arrow indicates the orientation of the retinas. T = temporal, V = ventral. Scale bars: 1 mm.

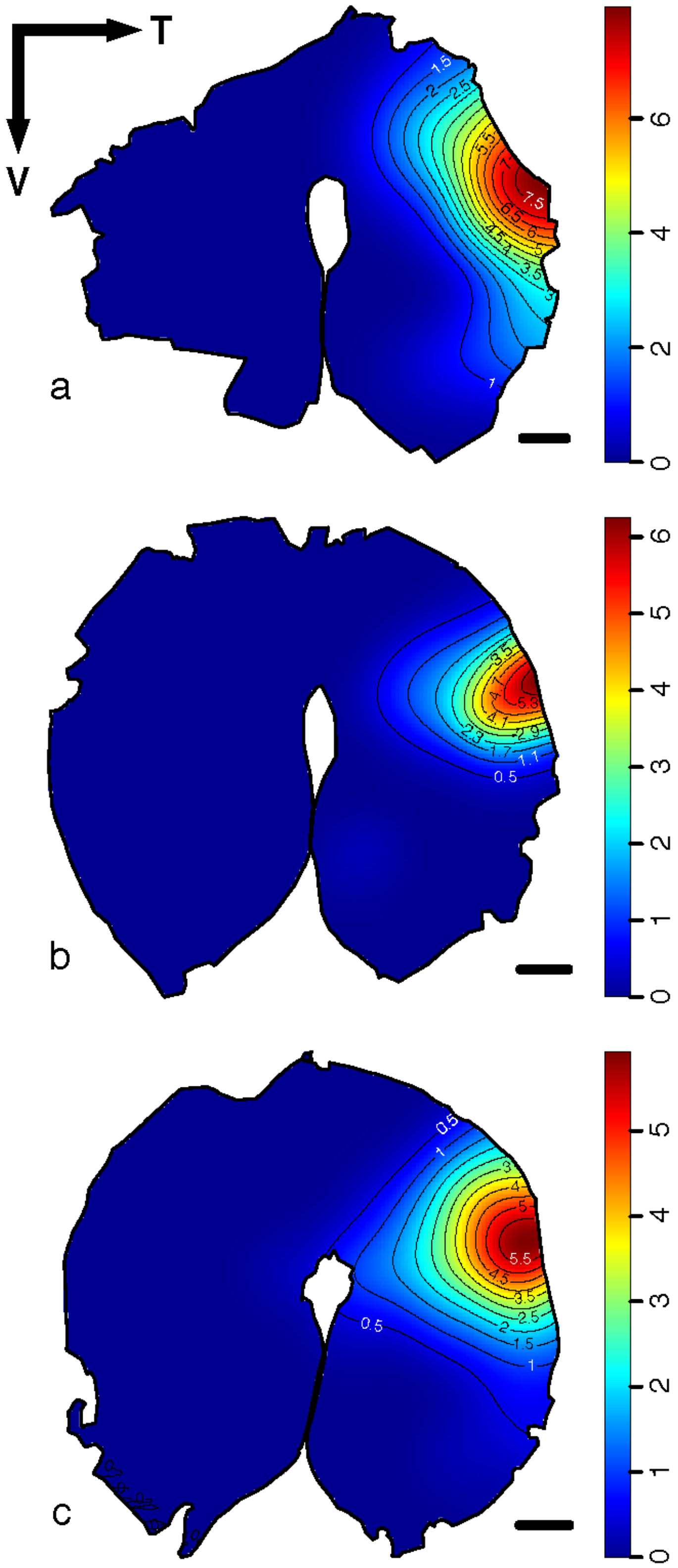


**Fig. S10:** Topographic distribution of SWS2B opsin densities in the retina of three individuals of *A. akindynos* of different sex/life stage: female (a), male (b) and immature (c). The black lines represent iso-density contours and values are expressed in densities x $10^3$ cells/$mm^3$. The black arrow indicates the orientation of the retinas. T = temporal, V = ventral. Scale bars: 1 mm.

**References**


1. Cortesi, F. *et al.* Ancestral duplications and highly dynamic opsin gene evolution in percomorph fishes. *Proc. Natl. Acad. Sci.* **112,** 1493–1498 (2015).
2. de Busserolles, F. *et al.* Pushing the limits of photoreception in twilight conditions: The rod-like cone retina of the deep-sea pearlsides. *Sci. Adv.* **3,** eaao4709 (2017).
3. Afgan, E. *et al.* Genomics Virtual Laboratory: A practical bioinformatics workbench for the cloud. *PLoS One* **10,** e0140829 (2015).
4. Coimbra, J. P., Nolan, P. M., Collin, S. P. & Hart, N. S. Retinal ganglion cell topography and spatial resolving power in penguins. *Brain. Behav. Evol.* **80,** 254–268 (2012).
5. Coimbra, J. P., Videira Marceliano, M. L., Da Silveira Andrade-Da-Costa, B. L. & Yamada, E. S. The retina of tyrant flycatchers: Topographic organization of neuronal density and size in the ganglion cell layer of the great kiskadee Pitangus sulphuratus and the rusty margined flycatcher Myiozetetes cayanensis (Aves: Tyrannidae). *Brain. Behav. Evol.* **68,** 15–25 (2006).
6. Ullmann, J. F. P., Moore, B. A., Temple, S. E., Fernández-Juricic, E. & Collin, S. P. The retinal wholemount technique: A window to understanding the brain and behaviour. *Brain. Behav. Evol.* **79,** 26–44 (2012).
7. Allison, W. T. *et al.* Ontogeny of cone photoreceptor mosaics in zebrafish. *J. Comp. Neurol.* **518,** 4182–4195 (2010).
8. Raymond, P. A. & Barthel, L. K. A moving wave patterns the cone photoreceptor mosaic array in the zebrafish retina. *Int. J. Dev. Biol.* **48,** 935–45 (2004).
9. Dalton, B. E., Loew, E. R., Cronin, T. W. & Carleton, K. L. Spectral tuning by opsin coexpression in retinal regions that view different parts of the visual field. *Proc. R. Soc. B Biol. Sci.* **281,** 20141980–20141980 (2014).
10. Slomianka, L. & West, M. J. Estimators of the precision of stereological estimates: An example based on the CA1 pyramidal cell layer of rats. *Neuroscience* **136,** 757–767 (2005).
11. Glaser, E. M. & Wilson, P. D. The coefficient of error of optical fractionator population size estimates: A computer simulation comparing three estimators. *J. Microsc.* **192,** 163–171 (1998).
12. Collin, S. P. & Collin, H. B. Topographic analysis of the retinal ganglion cell layer and optic nerve in the sandlanceLimnichthyes fasciatus (Creeiidae, Perciformes). *J. Comp. Neurol.* **278,** 226–241 (1988).
13. Hughes, A. A quantitative analysis of the cat retinal ganglion cell topography. *J. Comp. Neurol.* **163,** 107–128 (1975).
14. Wong, R. O. L. & Hughes, A. The morphology, number, and distribution of a large population of confirmed displaced amacrine cells in the adult cat retina. *J. Comp. Neurol.* **255,** 159–177 (1987).
15. Collin, S. P. & Pettigrew, J. D. Retinal ganglion cell topography in teleosts: A comparison between nissl-stained material and retrograde labelling from the optic nerve. *J. Comp. Neurol.* **276,** 412–422 (1988).
16. Bailes, H. J., Trezise, A. E. O. & Collin, S. P. The number, morphology, and distribution of retinal ganglion cells and optic axons in the Australian lungfish Neoceratodus forsteri (Krefft 1870). *Vis. Neurosci.* **23,** 257–273 (2006).
17. Collin, S. P. A web-based archive for topographic maps of retinal cell distribution in vertebrates: Invited Paper. *Clin. Exp. Optom.* **91,** 85–95 (2008).
18. Garza-Gisholt, E., Hemmi, J. M., Hart, N. S. & Collin, S. P. A comparison of spatial analysis methods for the construction of topographic maps of retinal cell density. *PLoS One* **9,** (2014).

19. Collin, S. P. & Pettigrew, J. D. Quantitative Comparison of the Limits on Visual Spatial Resolution Set by the Ganglion Cell Layer in Twelve Species of Reef Teleosts. *Brain. Behav. Evol.* **34,** 184–192 (1989).
20. Mattheissen, L. Ueber die Beziehungen, welche zwischen dem Brechungsindex des Kerncentrums der Krystalllinse und den Dimensionen des Auges bestehen. *Pflagers Arch.* **27,** 519–523 (1882).
21. Coimbra, J. P., Kaswera-Kyamakya, C., Gilissen, E., Manger, P. R. & Collin, S. P. The Retina of Ansorge ' s Cusimanse (Crossarchus ansorgei): Number, Topography and Convergence of Photoreceptors and Ganglion Cells in Relation to Ecology and Behavior. *Brain. Behav. Evol.* **86,** 79–93 (2015).
22. Hart, N. S. Microspectrophotometry of visual pigments and oil droplets in a marine bird, the wedge-tailed shearwater Puffinus pacificus: topographic variations in photoreceptor spectral characteristics. *J. Exp. Biol.* **207,** 1229–1240 (2004).
23. Chung, W. S. & Marshall, N. J. Comparative visual ecology of cephalopods from different habitats. *Proc. R. Soc. B Biol. Sci.* **283,** (2016).
24. MacNichol, E. F. A unifying presentation of photopigment spectra. *Vision Res.* **26,** 1543–1556 (1986).
25. Govardovskii, V. I., Fyhrquist, N., Reuter, T., Kuzmin, D. G. & Donner, K. In search of the visual pigment template. *Vis. Neurosci.* **17,** S0952523800174036 (2000).
26. Hart, N., Partridge, J. & Cuthill, I. I. Visual pigments, oil droplets and cone photoreceptor distribution in the european starling (Sturnus vulgaris). *J Exp Biol* **201 (Pt 9),** 1433–1446 (1998).
27. Yokoyama, S. Evolution of dim-light and color vision pigments. *Annu. Rev. Genomics Hum. Genet.* **9,** 259–82 (2008).
28. Spady, T. C. *et al.* Evolution of the cichlid visual palette through ontogenetic subfunctionalization of the opsin gene arrays. *Mol. Biol. Evol.* **23,** 1538–47 (2006).
29. Parry, J. W. L. *et al.* Mix and match color vision: tuning spectral sensitivity by differential opsin gene expression in Lake Malawi cichlids. *Curr. Biol.* **15,** 1734–9 (2005).
30. McFarland, W. N. & Loew, E. R. Ultraviolet visual pigments in marine fishes of the family pomacentridae. *Vision Res.* **34,** 1393–6 (1994).
31. Siebeck, U. E., Parker, A. N., Sprenger, D., Mäthger, L. M. & Wallis, G. A Species of Reef Fish that Uses Ultraviolet Patterns for Covert Face Recognition. *Curr. Biol.* **20,** 407–410 (2010).
32. Wald, G. Pigments of the rertina: II. Sea robin, sea bass, and scup. *J. Gen. Physiol.* **20,** 45–56 (1936).
33. Toyama, M. *et al.* Presence of Rhodopsin and Porphyropsin in the Eyes of 164 Fishes, Representing Marine, Diadromous, Coastal and Freshwater SpeciesA Qualitative and Comparative Study. *Photochem. Photobiol.* **84,** 996–1002 (2008).
34. Koshitaka, H., Kinoshita, M., Vorobyev, M. & Arikawa, K. Tetrachromacy in a butterfly that has eight varieties of spectral receptors. *Proc. R. Soc. B Biol. Sci.* **275,** 947–954 (2008).
35. Matsumoto, Y., Fukamachi, S., Mitani, H. & Kawamura, S. Functional characterization of visual opsin repertoire in Medaka (Oryzias latipes). *Gene* **371,** 268–278 (2006).
36. Hunt, D. M., Dulai, K. S., Partridge, J. C., Cottrill, P. & Bowmaker, J. K. The molecular basis for spectral tuning of rod visual pigments in deep-sea fish. *J. Exp. Biol.* **204,** 3333–44 (2001).
37. Takahashi, Y. & Ebrey, T. G. Molecular basis of spectral tuning in the newt short wavelength sensitive visual pigment. *Biochemistry* **42,** 6025–6034 (2003).

38. Dungan, S. Z., Kosyakov, A. & Chang, B. S. W. Spectral tuning of killer whale (*Orcinus orca*) rhodopsin: Evidence for positive selection and functional adaptation in a cetacean visual pigment. *Mol. Biol. Evol.* **33,** 323–336 (2016).
39. Yokoyama, S., Zhang, H., Radlwimmer, F. B. & Blow, N. S. Adaptive evolution of color vision of the Comoran coelacanth (Latimeria chalumnae). *Proc. Natl. Acad. Sci.* **96,** 6279–6284 (1999).
40. Wilkie, S. E. *et al.* Spectral tuning of avian violet- and ultraviolet-sensitive visual pigments. *Biochemistry* **39,** 7895–7901 (2000).
41. Yokoyama, S., Takenaka, N. & Blow, N. A novel spectral tuning in the short wavelength-sensitive (SWS1 and SWS2) pigments of bluefin killifish (Lucania goodei). *Gene* **396,** 196–202 (2007).
42. Lin, S. W. *et al.* Mechanisms of spectral tuning in blue cone visual pigments. Visible and raman spectroscopy of blue-shifted rhodopsin mutants. *J. Biol. Chem.* **273,** 24583–91 (1998).
43. Luehrmann, M. *et al.* Short term colour vision plasticity on the reef: Changes in opsin expression under varying light conditions differ between ecologically distinct reef fish species. *J. Exp. Biol.* (2018).
